# Comparative transcriptome analysis of peripheral blood mononuclear cells in hepatitis B-related acute-on-chronic liver failure

**DOI:** 10.1038/srep20759

**Published:** 2016-02-10

**Authors:** Qian Zhou, Wenchao Ding, Longyan Jiang, Jiaojiao Xin, Tianzhou Wu, Dongyan Shi, Jing Jiang, Hongcui Cao, Lanjuan Li, Jun Li

**Affiliations:** 1 State Key Laboratory for Diagnosis and Treatment of Infectious Diseases, Collaborative Innovation Center for Diagnosis and Treatment of Infectious Diseases, The First Affiliated Hospital, Zhejiang University School of Medicine, 79 Qingchun Rd., Hangzhou, 310003 China

## Abstract

Analysis of the transcriptome of peripheral blood mononuclear cells (PBMCs) from patients with hepatitis B-related acute-on-chronic liver failure (HBV-ACLF) is essential to elucidate the pathogenesis of HBV-ACLF and identify HBV-ACLF-specific biomarkers. In this study, high-throughput sequencing was performed to characterize the transcriptome of PMBCs from patients with HBV-ACLF. Specifically, 2381 differentially expressed genes (DEGs) and 776 differentially expressed transcripts were identified through comparisons with patients with chronic hepatitis B (CHB) and healthy controls. Gene Ontology (GO) analysis identified 114 GO terms that were clustered into 12 groups. We merged 10 dysregulated genes selected from these grouped GO terms and non-clustered terms with four significant genes with a specificity of >0.8 in the HBV-ACLF patients to obtain a set of 13 unique genes. The quantitative real-time polymerase chain reaction (qRT-PCR) validation of the top six genes (CYP19A1, SEMA6B, INHBA, DEFT1P, AZU1 and DEFA4) was consistent with the results of messenger ribonucleic acid (mRNA) sequencing. A further receiver operating characteristic (ROC) analysis revealed that the areas under the ROC curves of the six genes were all >0.8, which indicated their significant diagnostic potentials for HBV-ACLF. Conclusion: The transcriptome characteristics of PBMCs are altered in patients with HBV-ACLF, and six genes may serve as biomarkers of HBV-ACLF.

Acute-on-chronic liver failure (ACLF) is an acute deterioration of pre-existing chronic liver diseases that ultimately results in increased mortality due to multisystem organ failure. This failure is usually induced by a precipitating event^1^,^2^. In most Asian countries, the aetiology of ACLF is primarily due to hepatitis B virus (HBV) infection because of the high prevalence of this virus^3^. Establishment of specific and sensitive biomarkers for early diagnosis is critical to identify patients who require early treatment. Genome-wide gene expression profiling is extensively used to discover new potential biomarkers for the diagnosis or prediction of disease severity and identification of novel drug targets. However, tissue sampling has limited the accurate investigation of the pathogenesis of HBV-ACLF. Specifically, patients experiencing severe hepatic failure may not be able to undergo a biopsy. Therefore, the use of blood as a surrogate tissue, which can be obtained with a minimally invasive procedure, is an attractive alternative to hepatic biopsies. Many studies have indicated that the messenger ribonucleic acid (mRNA) expression levels of specific genes in peripheral blood mononuclear cells (PBMCs) can serve as a signature of specific diseases, including Parkinson’s disease^4^, cancer^5^, leukaemia^6^, and heart failure^7^. Recently, many genes have also been identified for the diagnosis and prognostic prediction of the progression of HBV-ACLF^8^,^9^,^10^. However, these biomarkers lack sensitivity and specificity. Previous studies have indicated that almost 90% of multi-exon human genes undergo alternative splicing during development, cell differentiation, and disease progression^11^. Transcriptome analyses of PBMCs have been successfully applied to numerous complex diseases, such as cardiovascular diseases^12^ and chronic respiratory diseases^13^. We hypothesized that the pathological process of HBV-ACLF changes the transcriptome profile of PBMCs. In this study, we characterized the transcriptome profile of PBMCs in patients with HBV-ACLF using high-throughput sequencing (HTS) to detect alterations present during disease progression and validated the identified differentially expressed genes (DEGs) via quantitative real-time polymerase chain reaction (qRT-PCR).

## Results

### Clinical characteristics of the enrolled subjects

A total of 24 patients with HBV-ACLF, 24 patients with chronic hepatitis B (CHB) and 24 healthy controls were enrolled in this study, 12 of whom were placed in a sequencing group (4 subjects each from the HBV-ACLF, CHB and healthy groups) and 60 of whom were assigned to a validation group (20 subjects per group). Patients with concomitant diseases were excluded. All of the HBV-ACLF patients were diagnosed with cirrhosis, and all of the CHB patients were outpatients who were in the inactive disease phase. The HBV genotype was B in most of the patients, except for 8 without sufficient clinical data. Table 1 shows the demographic and clinical characteristics of the HBV-ACLF patients in the sequencing and validation groups at admission, as well as those of the CHB patients and healthy controls, and detailed information on these enrolled subjects is provided in Supplemental Tables S1 and S2. The demographic and clinical characteristics of the subjects in the sequencing group were similar to those of the subjects in the validation group. The distributions of age and gender did not differ between the HBV-ACLF and CHB groups (P > 0.05). The levels of alanine aminotransferase (ALT), aspartate aminotransferase (AST), and total bilirubin (TB) and the international normalized ratio (INR) in the HBV-ACLF patients were significantly different between the sequencing and validation groups (Table 1, P < 0.05). These significant differences observed may have been due to the small size of the sequencing group.

### Qualitative analysis of the PBMC transcriptomes of patients with HBV-ACLF and CHB and healthy controls

To identify changes in the transcriptome profile associated with HBV-ACLF, the transcriptomes of PMBCs from HBV-ACLF patients, CHB patients and healthy controls were characterized using HTS. Each group consisted of 4 subjects, as detailed in Supplemental Table S1. The number of sequencing paired-end reads for each sample ranged from 17.2 million to 28.5 million, and an average of 92.9% of reads from each sample were uniquely mapped to the human genome. Specifically, 30,893, 31,302 and 30,904 genes were identified (fragments per kb of exon per million mapped reads (FPKM) >0) in the HBV-ACLF, CHB and healthy groups, respectively. Detailed information regarding each sample was also recorded (Table 2).

### Detection of DEGs in HBV-ACLF patients

**Figure 1:**
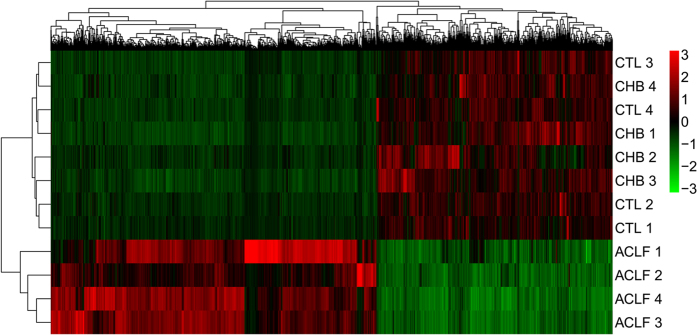
The clustering results for the samples and mRNAs, as determined using the normalized expression of 2381 aberrantly expressed mRNAs in the HBV-ACLF, CHB and CTL subjects. The samples are labelled as follows: ACLF, patients with HBV-ACLF; CHB, patients with CHB; CTL, healthy controls.

The differential expression of genes in the transcriptome of patients with HBV-ACLF compared with CHB patients and healthy controls was analysed using the Cufflinks software package (free software, version 2.2.1)^14^. This analysis indicated that 3079 and 2763 genes were significantly differentially expressed in patients with HBV-ACLF compared with CHB patients and healthy controls, respectively. However, only 36 genes were significantly differentially expressed between CHB patients and healthy controls. The DEGs from the previous first two groups were intersected to obtain a set of 2381 common DEGs in the patients with HBV-ACLF, of which 1380 were upregulated and 1001 of which were downregulated (Supplemental Table S3). Clustering analysis based on the normalized expression of these 2381 genes revealed that all of the patients with HBV-ACLF were clustered together, which indicated that these DEGs can be used to distinguish patients with HBV-ACLF from those with CHB and healthy controls (Fig. 1).

### Gene Ontology (GO) analysis

**Figure 2:**
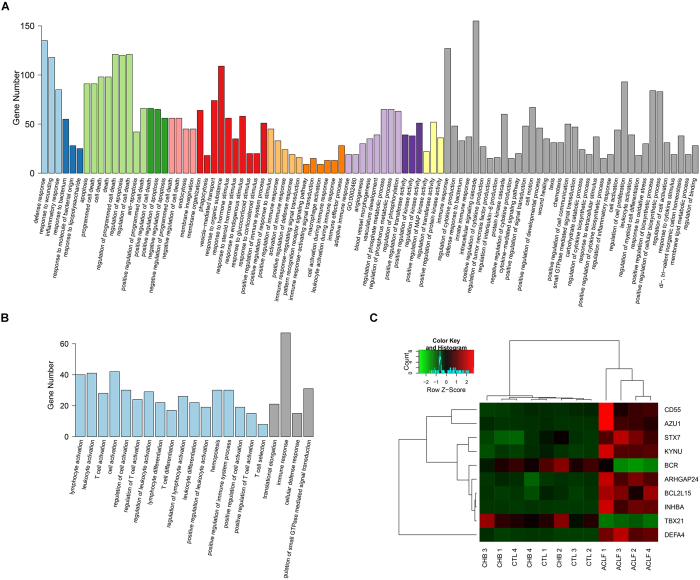
(**A**) GO terms of the 1380 upregulated genes. (**B**) GO terms of the 1001 downregulated genes. (**C**) Clustering analysis of 10 genes that distinguished patients with HBV-ACLF from those with CHB and the healthy controls. The samples are labelled as follows: ACLF, patients with HBV-ACLF; CHB, patients with CHB; CTL, healthy controls.

To study the roles and functions of these gene products, the biological processes that these DEGs participate in were identified by GO enrichment analysis using DAVID^15^. In total, 114 GO terms were identified, as detailed in Fig. 2, including the immune response, the regulation of the immune system, the regulation of cell death, haematopoiesis, the defence response, the regulation of kinase activity, and immune cell activation, among others. Of these processes, 93 terms were identified based on the upregulated genes (Fig. 2A), and the other 21 terms were identified based on the downregulated genes (Fig. 2B). These terms were then clustered into 11 clusters and 1 cluster, respectively. However, 36 terms in the upregulated group and 4 terms in the downregulated group did not belong to any of the clusters.

### Identification of representative and specific genes in HBV-ACLF patients

Based on the results of the GO analysis, the significantly differentially expressed genes that underwent the largest fold changes in expression between patients with HBV-ACLF or CHB were selected from each cluster of GO terms and the group of all non-clustered terms for further analysis. After elimination of redundancy, ten unique representative genes were identified: INHBA, AZU1, DEFA4, KYNU, CD55, ARHGAP24, STX7, BCR, TBX21, and BCL2L15. Clustering analysis using these 10 genes was also able to distinguish the HBV-ACLF group from the CHB and healthy groups (Fig. 2C). Additionally, the following four genes with a specificity >0.8 were identified in the HBV-ACLF patients: CYP19A1, SEMA6B, INHBA and TEFT1P. These genes were specifically expressed in the patients with HBV-ACLF but were minimally expressed in those with CHB and in the healthy controls. We combined the representative and specific genes to obtain a set of 13 genes (Table 3) that may serve as a signature of HBV-ACLF.

### Validation of the mRNA expression levels of six genes using qRT-PCR

**Figure 3:**
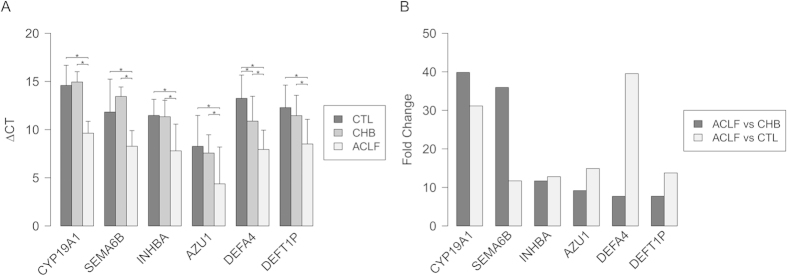
(**A**) The ΔCT results of the top 6 DEGs identified using qRT-PCR. (**B**) Comparison of the fold changes of the top 6 DEGs identified using qRT-PCR between the HBV-ACLF patients, CHB patients and healthy controls (CTL).

To identify the optimal biomarkers for the diagnosis and prognosis of HBV-ACLF, the top 6 genes with fold changes of >20.0 were selected for further qRT-PCR validation. Samples from another 60 patients (20 per group) were subjected to qRT-PCR, as detailed in Supplemental Table S2. The qRT-PCR data indicated that all six genes were significantly upregulated (Fig. 3), which was consistent with the results of the initial screening analysis. Specifically, the expression levels of these 6 genes were increased >7.0-fold in patients with HBV-ACLF compared with patients with CHB: CYP19A1 (39.8-fold), SEMA6B (35.9-fold), INHBA (11.7-fold), AZU1 (9.2-fold), DEFT1P (7.7-fold) and DEFA4 (7.7-fold).

### Receiver operating characteristic (ROC) analysis of the selected top 6 DEGs

**Figure 4:**
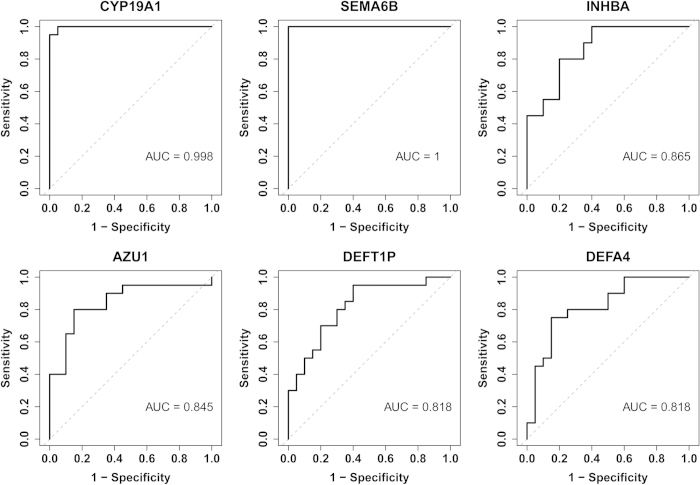
ROC curves for the top 6 DEGs: CYP19A1, SEMA6B, INHBA, DEFT1P, AZU1, and DEFA4.

To investigate the expression levels of CYP19A1, SEMA6B, INHBA, DEFT1P, AZU1 and DEFA4 as diagnostic or prognostic biomarkers of HBV-ACLF, the qRT-PCR data from 40 patients with HBV-ACLF and CHB (20 per group) were subjected to ROC analysis, which indicated that these markers were reliably predictive. As shown in Fig. 4, the areas under the ROCs (AUROCs) of the 6 genes were all >0.8, especially for CYP19A1 and SEMALB, which yielded AUROCs of 0.998 and 1, respectively. These results indicate that the expression levels of these 6 DEGs are highly specific and may serve as sensitive biomarkers for predicting HBV-ACLF.

### Identification of the differentially expressed transcripts between HBV-ACLF and CHB

To identify differences in the splice ratios between HBV-ACLF and CHB patients, we also employed Cufflinks and cummeRbund to investigate the expression of alternatively spliced transcripts. A differential analysis of alternatively spliced transcripts revealed that 776 transcripts, which were generated from 729 genes, were significantly and differentially expressed. Among these genes, 27 genes that correspond to 27 transcripts were not differentially expressed between HBV-ACLF and CHB, whereas the other genes were differentially expressed (Supplemental Table S4). Furthermore, we selected the top 10 upregulated and downregulated transcripts according to the average fold change and found that their corresponding genes were also differentially expressed between HBV-ACLF and CHB (Supplemental Table S5). However, these genes were not necessarily the most differentially expressed genes in each cluster according to the GO analysis results.

## Discussion

ACLF usually develops on a background of established cirrhosis following a precipitating event. The best-studied precipitating events are surgery and superimposed viral hepatitis, and HBV infection accounts for the majority of aetiologies^1^,^3^. However, the possible mechanisms underlying HBV-ACLF have not yet been elucidated. Recently, an increasing number of studies have indicated that unregulated inflammation rather than a direct viral cause may be the major contributing factor in the development of HBV-ACLF^16^. Furthermore, both the severity of inflammation and the occurrence of new infection are associated with a higher risk of death in ACLF patients^17^,^18^. Previous studies have demonstrated that the differentially expressed AZU1, DEFT1P and DEFA4 genes are involved in the defence response^19^,^20^,^21^. Combined with the ROC analysis results, these data tentatively indicate that new, severe infections and their associated systemic inflammatory responses in HBV-ACLF patients may be responsible for pathophysiological development.

Recently, more than 20 different biomarkers, including TB, INR, Cr, PT, ALB, AST, ALT, BUN, ammonia, macrophage inflammatory protein 3α, haemoglobin, electrolyte disturbances and thymosin β4, have been reported to be related to the severity and mortality of ACLF^12^,^22^. However, no study has reported on the expression of the six genes CYP19A1, SEMA6B, INHBA, DEFT1P, AZU1 and DEFA4 in HBV-ACLF. In this work, the DEGs between patients with HBV-ACLF and CHB and healthy controls were initially screened using HTS. A qRT-PCR validation then demonstrated that the levels of CYP19A1 and SEMA6B expression were significantly increased in the patients with HBV-ACLF compared with those with CHB. CYP19A1 reportedly encodes aromatase, which is a key enzyme responsible for a critical step in the catalysis of many reactions involved in drug metabolism and synthesis of cholesterol, steroids and protein. Moreover, these biochemical reactions are primarily regulated by the liver. Therefore, dysregulation of CYP19A1 may partly reflect liver malfunction. In addition, previous studies have established that SEMA6B is involved in cancer progression and highly expressed in cancer tissues^23^,^24^. However, the expression of SEMA6B in the PBMCs of patients with HBV-ACLF has not yet been investigated. The upregulation of this gene in HBV-ACLF may also contribute to tumourigenesis in damaged livers. Specifically, an ROC analysis of CYP19A1 and SEMA6B indicated that these genes could distinguish HBV-ACLF from CHB with high sensitivity and specificity. Therefore, these genes may serve as biomarkers for early diagnosis of HBV-ACLF.

Inhibin β A (INHBA) is a ligand in the transforming growth factor β (TGF-β) superfamily whose biological functions have been reported in many studies; they include neoplastic progression, development, proliferation and immune regulation. In addition, overexpression of INHBA has been established to be correlated with poor prognosis based on an analysis of the expression level and functional impact of INHBA in lung adenocarcinoma^25^, colorectal cancer^26^, gastric cancer^27^, prostate cancer^28^, oesophageal adenocarcinoma^29^, and other diseases. Because this gene was highly expressed in the PBMCs of patients with HBV-ACLF and had a high AUROC value (0.865), our study suggests that INHBA may serve as an appropriate prognostic biomarker of HBV-ACLF. Nevertheless, further investigations are needed to fully clarify this finding.

Preliminary analysis of the transcriptome profiles of PBMCs from patients with HBV-ACLF and CHB identified transcripts generated from non-DEGs via alternative splicing during the progression of HBV-ACLF that were differentially expressed. These data tentatively indicate that transcriptome analyses may feasibly identify the underlying genes involved in the diagnosis of HBV-ACLF that are non-differentially expressed or are differentially expressed with a low fold change.

Our previous study has demonstrated that the miRNA expression profile of PBMCs is altered in patients with HBV-ACLF^30^. We compared the 2381 DEGs in this study with the 2162 targeted genes of the 8 differentially expressed miRNAs in a previous study by integrating mRNA and miRNA analysis and revealed a set of 300 common genes. Of these 300 genes, only one gene, ARHGAP24, which is involved in cell migration, was observed among the 13 significantly differentially expressed genes detected in this study. The detailed relationship between miRNA and mRNA expression in HBV-ACLF needs to be further clarified.

In summary, our transcriptome analysis of PBMCs from HBV-ACLF patients has revealed the presence of alterations, and further qRT-PCR validation and ROC analysis have indicated that six genes are differentially expressed between patients with HBV-ACLF and CHB (CYP19A1, SEMA6B, INHBA, DEFT1P, AZU1 and DEFA4). These genes are potential biomarkers with high sensitivity and specificity for HBV-ACLF diagnosis and prognosis. To permit the clinical application of these biomarkers, a larger cohort of HBV-ACLF patients needs to be further validated using HTS. Whether the HBV genotype and HBV mutation status affect the progression and transcriptome profile of CHB patients also need to be clarified in a future study.

## Methods

### Patient information

All patients enrolled in the study provided written informed consent. The protocol of this study was approved by the Clinical Research Ethics Committee of the First Affiliated Hospital at the Zhejiang University School of Medicine. The experiment methods were carried out in accordance with the approved guidelines. Four subjects in each group (HBV-ACLF, CHB and healthy control) were enrolled for the initial HTS analysis. Another 60 subjects (20 subjects per group) were enrolled for validation purposes. HBV-ACLF was diagnosed based on the CLIF-SOFA scoring system by addressing six functional failures (hepatic, renal, cerebral, coagulatory, circulatory and respiratory). Each failure was weighted in terms of severity from 0 to 4 to yield a total score between 0 and 24^31^. Cirrhosis was diagnosed based on clinical and diagnostic findings from laboratory tests, endoscopy and radiologic imaging. The liver function indexes, including the ALB, ALT, AST, TB, and sodium levels, INR, the HBV DNA copy number and the model for end-stage liver disease (MELD) score^32^, were recorded. The time of admission to the hospital was considered to be time zero. The CHB patients were enrolled based on the following criteria from the 2009 AASLD guidelines^33^: Hepatitis B surface antigen (HBsAg) positive >6 months, serum HBV DNA >10^5^ copies/ml, and persistent or intermittent elevations in ALT/AST levels indicative of chronic hepatitis. The following patients were excluded: 1) those infected with other hepatitis viruses (including A, C, D and E); 2) those with superimposed viral infections; and 3) those with hypertension, diabetes, carcinoma, or severe intrinsic renal disease. All patients received standard medical therapy during hospitalization, including high-caloric nutrition, lactulose for hepatic encephalopathy, renal replacement for hepatorenal syndrome and uraemic symptoms, and prophylactic antibiotics (quinolones) for the primary prevention of spontaneous bacterial peritonitis^34^. Twenty-four healthy adults were enrolled as normal controls, including four individuals for the initial screening of DEGs. The clinical characteristics of the enrolled patients and normal subjects are presented in Table 1, Supplemental Tables S1 and S2.

### RNA extraction, sequencing library preparation and next-generation sequencing

Citrate-anticoagulated peripheral blood was obtained from all subjects upon admittance to the hospital prior to treatment. Ficoll-Histopaque (Sigma Aldrich, MO, USA) was used for the PBMC collection. The freshly isolated PBMC samples were suspended in TRIzol reagent (Invitrogen, CA, USA), and the total RNA was immediately extracted according to the manufacturer’s instructions. The freshly extracted total RNA was stored at −80 °C for subsequent testing. The sequencing library was prepared using the Illumina^®^ TruSeq™ RNA Sample Preparation Guide, which consisted of purification of poly-A containing mRNA molecules, fragmentation of mRNA, reverse transcription, end repair, addition of a single ‘A’ base, ligation of the adapters, and purification and enrichment with PCR. The library was sequenced using an Illumina HiSeq 2000. The average number of sequencing paired-end reads (2 × 101bp) was approximately 22 million.

### Bioinformatics analysis of the sequencing data

The sequencing reads were mapped against hg38 using STAR (v2.4.0), which is an ultrafast universal RNA-seq aligner^35^. All parameters were set to the default values except for the allowed maximum mismatch, which was set to 5% for each read, and the output of the BAM files, which were sorted by coordinate. The transcripts were assembled *de novo* using Cufflinks (v2.2.1)^13^, and the novel built GTF file was merged with the GTF file of hg38. With the merged GTF file, the FPKM of each transcript was estimated using the cuffquant and cuffnorm tools contained in the Cufflinks suite. The differential expression of each transcript and each gene was analysed using cuffdiff, which is also a component of Cufflinks. Condition-specific genes were also identified with cummeRbund by estimating the Jensen-Shannon distance. The significantly upregulated or downregulated genes in HBV-ACLF were then submitted to The Database for Annotation, Visualization and Integrated Discovery (DAVID, v6.7)^14^ for GO enrichment analyses. The enriched GO terms with a false discovery rate (FDR) of <0.05 were considered significant and further subjected to a cluster analysis.

### qRT-PCR

The DEGs identified based on the initial HTS results were validated in another 60 subjects (20 subjects per group) using qRT-PCR. The top 6 genes (CYP19A1, SEMA6B, INHBA, DEFT1P, AZU1, and DEFA4) from the aforementioned set of genes whose expression level increased >20-fold were selected for further validation. Total RNA was extracted from PBMCs and reverse transcribed. Complementary DNA (cDNA) was synthesized using a QuantiTect Reverse Transcription kit (Qiagen, CA, USA), and qRT-PCR reactions were performed with Platinum SYBR Green qPCR SuperMix UDG (Invitrogen, CA, USA) using specific gene primers (Sangon, Shanghai, China) following the manufacturer’s instructions. The following primers were used: CYP19A1, 5′-acatctggacaggttggagg-3′ and 5′-ttgatgaggagagcttgcca-3′; SEMA6B, 5′-ctctttgtgtgcggttccaa-3′ and 5′-gagcatcccgtcagagaaga-3′; INHBA, 5′-tgtacccaactctcagccag-3′ and 5′-tgccctccttccaatgtcat-3′; DEFT1P, 5′-ccctcttcactctgcctacc-3′ and 5′-gctgagtctgaaagcggaag-3′; AZU1, 5′-gaacctgaacgacctgatgc-3′ and 5′-tgacaaacctgggaaaacgg-3′; DEFA4, 5′-aagtcctctcctctgtgtgc-3′ and 5′-ttgatgaggagagcttgcca-3′. Thermal cycling was performed using a Bio-Rad DNA Engine Opticon-2 (Bio-Rad, CA, USA) as follows: 50 °C for 2 min, 95 °C for 30 s, and 40 cycles of 5 s at 95 °C and 34s at 62 °C. The target genes were assayed in triplicate on each plate. Glyceraldehyde phosphate dehydrogenase (GAPDH) was used as a housekeeping gene to normalize and evaluate each target gene on the same plate for data comparison.

### Statistical analyses

All statistical analyses were performed with the R software package. The significant DEGs and transcripts were analysed using the Cuffdiff and cummeRbund package^36^. Unsupervised hierarchical clustering of the samples from the different groups with significant DEGs was performed using the ‘heatmap.2’ function contained in the gplots package^37^. The DEGs validated via qRT-PCR were identified using Student’s t-test. ROC analysis was performed using the pROC package^38^. A P-value of <0.05 was considered to indicate a significant difference.

## Additional Information

**How to cite this article**: Zhou, Q. *et al*. Comparative transcriptome analysis of peripheral blood mononuclear cells in hepatitis B-related acute-on-chronic liver failure. *Sci. Rep.* **6**, 20759; doi: 10.1038/srep20759 (2016).

## Supplementary Material

Supplementary Information

## Figures and Tables

**Table 1:** The demographic and clinical characteristics of the enrolled patients and healthy subjects.

Clinical parameter	Sequencing group (n = 4 per group)			Validation group (n = 20 per group)			
	HBV-ACLF	CHB	Healthy	HBV-ACLF	CHB	Healthy	P-value^*^
Age (years)	41.0 ± 12.1	40.8 ± 7.9	31.3 ± 7.3	45.0 ± 9.6	41.3 ± 11.1	28.5 ± 3.5	0.472
Sex (M/F)	4/0	4/0	1/3	16/4	12/8	10/10	0.807
ALB (g/L)	33.9 ± 3.6	45.9 ± 1.3	49.5 ± 3.1	30.8 ± 4.2	49.3 ± 2.1	47.6 ± 2.5	0.188
ALT (U/L)	69.5 ± 20.5	26.3 ± 9.1	15.8 ± 4.1	389.9 ± 286.7	28.6 ± 15.3	14.7 ± 9.5	< 0.001
AST (U/L)	105.3 ± 34.2	24.0 ± 2.4	17.3 ± 2.1	328.4 ± 244.4	27.3 ± 12.2	16.7 ± 3.4	<0.001
TB (μmol/L)	529.3 ± 157.5	14.5 ± 5.1	11.3 ± 2.2	361.8 ± 108.4	17.0 ± 6.7	18.3 ± 16.8	0.015
Cr (μmol/L)	82.3 ± 68.3	79.8 ± 24.5	59.8 ± 25.7	70.9 ± 22.7	65.5 ± 13.2	62.6 ± 18.8	0.054
Sodium (μmol/L)	133.8 ± 2.9	141.5 ± 1.3	139.8 ± 1.0	137.1 ± 3.1	140.9 ± 1.6	139.6 ± 1.1	0.065
INR	1.6 ± 0.3	1.1 ± 0.1	NA	2.1 ± 0.4	1.0 ± 0.1	NA	0.027
HBV-DNA log_10_ (IU/ml)	5.9 ± 1.2	5.8 ± 1.2	NA	5.6 ± 1.3	7.3 ± 1.2	NA	0.757
MELD	24.4 ± 2.8	7.1 ± 1.7	NA	26.3 ± 2.6	5.7 ± 2.2	NA	0.220

^*^HBV-ACLF patients between the sequencing and validation groups.

**Table 2:** Qualitative analysis results of transcriptomes of PBMCs from patients with HBV-ACLF and CHB and healthy controls.

Sample	Total reads (PE)	Uniquely mapped reads	Uniquely mapped percentage (%)	Gene number
HBV-ACLF 1	21,367,886	19,768,639	92.52	27173
HBV-ACLF 2	23,775,867	21,079,403	88.66	25882
HBV-ACLF 3	20,791,403	19,122,098	91.97	26035
HBV-ACLF 4	17,184,481	15,848,096	92.22	26084
CHB 1	22,446,813	21,002,998	93.57	26390
CHB 2	22,640,366	21,135,131	93.35	26465
CHB 3	26,577,273	24,931,772	93.81	27202
CHB 4	20,997,156	19,662,343	93.64	26117
CTL 1	28,515,761	26,712,276	93.68	25757
CTL 2	19,305,248	18,080,128	93.65	26832
CTL 3	19,531,539	18,283,564	93.61	26088
CTL 4	20,559,393	19,238,630	93.58	25109

**Table 3:** List of the top 13 upregulated and downregulated DEGs (P < 0.001) identified by HTS analysis.

Gene	Description	Fold Change (HTS)	
		ACLF vs. CHB	ACLF vs. CTL
CYP19A1	cytochrome P450, family 19, subfamily A, polypeptide 1	82.7	55.9
SEMA6B	sema domain, transmembrane domain (TM), and cytoplasmic domain, (semaphorin) 6B	40.5	41.3
INHBA	inhibin, beta A	38.2	77.6
DEFTIP	defensin, theta 1 pseudogene	34.3	21.4
AZU1	azurocidin 1	31.0	35.2
DEFA4	defensin, alpha 4, corticostatin	28.5	50.7
BCL2L15	BCL2-like 15	7.0	5.6
KYNU	kynureninase	3.5	3.3
CD55	CD55 molecule, decay accelerating factor for complement	3.4	3.0
ARHGAP24	Rho GTPase activating protein 24	3.3	2.9
STX7	syntaxin 7	1.5	1.6
BCR	breakpoint cluster region	0.6	0.6
TBX21	T-box 21	0.2	0.2
