# Supplementary material for: Comparative transcriptome analysis of peripheral blood mononuclear cells in hepatitis B-related acute-on-chronic liver failure
Source: Sci Rep. 2016 Feb 10;6:20759. doi: 10.1038/srep20759 (PMC4748289; doi:10.1038/srep20759)
Supplement: Supplementary Information [file srep20759-s1.pdf]

Comparative transcriptome analysis of peripheral blood mononuclear cells in hepatitis B-related acute-on-chronic liver failure

Qian Zhou, Wenchao Ding, Longyan Jiang, Jiaojiao Xin, Tianzhou Wu, Dongyan Shi, Jing Jiang, Hongcui Cao, Lanjuan Li, Jun Li

Contents

Supplemental Table S1.

Supplemental Table S2.

Supplemental Table S4.

Supplemental Table S5.

Supplemental Table S1. The clinical characteristics of the patients in the sequencing group.

Patients	Age (years)	Sex (F/M)	ALB (g/L)	ALT (U/L)	AST (U/L)	TB (μmol/L)	Cr (μmol/L)	Sodium (μmol/L)	INR	HBV-DNA log10(IU/ml)	MELD
Healthy-1	26	F	54.1	13	19	12	40	139	1.05	NA	5.64
Healthy-2	28	F	48.3	21	16	14	46	140	1.11	NA	6.84
Healthy-3	29	F	47.2	12	15	9	56	139	NA	NA	NA
Healthy-4	42	M	48.4	17	19	10	97	141	NA	NA	NA
CHB-1	51	M	46.4	28	26	18	73	141	1.13	7.10	7.99
CHB-2	41	M	45.6	15	26	19	106	140	1.02	4.27	8.78
CHB-3	39	M	47.2	37	23	13	49	143	0.96	5.86	4.94
CHB-4	32	M	44.2	25	21	8	91	142	1.3	5.80	6.76
ACLF-1	48	M	38.4	94	112	511	67	133	1.36	6.44	22.72
ACLF-2	54	M	35.1	44	55	314	42	138	1.41	5.12	21.28
ACLF-3	28	M	31.6	68	128	630	14	132	1.71	7.33	26.07
ACLF-4	34	M	30.5	72	126	662	59	132	1.95	4.61	27.73

Supplemental Table S2. The clinical characteristics of the patients in the validation group.

Patients	Age (years)	Sex (F/M)	ALB (g/L)	ALT (U/L)	AST (U/L)	TB (μmol/L)	Cr (μmol/L)	Sodium (μmol/L)	INR	HBV-DNA log10(IU/ml)	MELD
Healthy-5	34	M	48.1	47	26	79	11	NA	NA	NA	NA
Healthy-6	30	F	47.2	12	15	9	56	139	NA	NA	NA
Healthy-7	27	F	40.9	6	12	8	56	142	NA	NA	NA
Healthy-8	27	F	48.5	9	16	10	67	139	NA	NA	NA
Healthy-9	31	F	47.5	11	15	20	68	140	NA	NA	NA
Healthy-10	29	M	47.7	15	16	7	77	140	NA	NA	NA
Healthy-11	27	F	49.6	9	NA	10	60	NA	NA	NA	NA
Healthy-12	32	F	44.5	10	16	6	48	138	NA	NA	NA
Healthy-13	33	F	49.0	12	15	17	66	139	NA	NA	NA
Healthy-14	20	M	46.5	20	19	29	41	138	NA	NA	NA
Healthy-15	26	M	49.7	7	14	23	80	140	NA	NA	NA
Healthy-16	30	M	45.6	13	19	15	70	141	NA	NA	NA
Healthy-17	27	F	49.8	11	19	14	50	140	NA	NA	NA
Healthy-18	25	F	44.3	10	14	6	52	139	NA	NA	NA
Healthy-19	27	M	48.6	17	18	6	78	139	NA	NA	NA
Healthy-20	24	M	49.0	23	18	23	65	139	NA	NA	NA
Healthy-21	33	M	49.0	14	19	22	81	141	NA	NA	NA
Healthy-22	31	M	51.6	29	NA	40	85	NA	NA	NA	NA
Healthy-23	30	M	49.9	12	NA	10	94	NA	NA	NA	NA
Healthy-24	27	F	45.5	6	12	12	46	140	NA	NA	NA
CHB-5	65	F	47.5	45	29	13	54	140	0.91	5.90	4.34
CHB-6	69	F	49.2	23	37	29	66	146	1.05	7.45	8.97
CHB-7	48	M	48.1	22	21	20	70	141	1.01	6.79	7.13

CHB-8	41	M	48.8	36	23	19	70	139	0.98	5.03	6.60
CHB-9	40	M	49.2	19	18	21	75	140	1.06	8.11	7.86
CHB-10	59	F	45.5	20	27	23	56	142	1.04	8.32	7.99
CHB-11	39	M	50.2	33	27	17	85	142	1.00	5.63	6.41
CHB-12	33	F	49.6	14	23	9	55	140	0.95	6.15	3.43
CHB-13	35	F	50.9	26	23	7	46	140	0.93	7.00	2.24
CHB-14	33	M	52.4	37	28	17	79	141	0.97	6.79	6.07
CHB-15	36	M	50.0	24	21	26	91	140	0.92	7.62	7.35
CHB-16	32	M	45.9	19	17	21	66	138	0.94	8.38	6.51
CHB-17	37	F	49.2	74	45	13	45	141	1.01	8.71	5.51
CHB-18	40	F	46.2	27	28	5	56	140	0.93	7.48	0.97
CHB-19	41	M	50.0	20	24	12	78	141	0.88	8.37	3.66
CHB-20	44	M	51.0	9	21	8	70	142	0.93	8.03	2.75
CHB-21	31	M	50.0	28	24	19	61	142	0.97	9.61	6.49
CHB-22	31	F	47.0	56	71	15	47	140	0.94	5.63	5.24
CHB-23	40	M	53.0	24	20	19	79	142	1.02	6.17	7.05
CHB-24	31	M	52.1	15	19	27	61	141	0.95	8.61	7.58
HBV-ACLF-5	60	F	22.9	462	562	302	107	138	2.65	5.00	30.02
HBV-ACLF-6	30	M	36.4	544	460	492	59	136	1.68	6.58	24.94
HBV-ACLF-7	41	M	30.8	71	58	360	67	135	1.90	NA	25.14
HBV-ACLF-8	47	M	36.1	452	515	273	59	136	2.16	6.43	25.53
HBV-ACLF-9	47	M	28.8	104	145	256	71	130	2.09	5.88	24.92
HBV-ACLF-10	36	M	29.8	85	88	478	45	135	1.90	6.47	26.21
HBV-ACLF-11	35	M	38.6	470	170	336	66	140	1.74	5.12	23.89
HBV-ACLF-12	39	M	31.6	1105	664	366	62	134	2.26	4.21	27.14
HBV-ACLF-13	52	M	36.3	244	188	646	100	132	1.66	6.08	27.00
HBV-ACLF-14	58	F	28.1	521	441	200	58	140	1.90	5.77	22.91
HBV-ACLF-15	47	F	26.9	331	910	350	47	139	2.25	4.27	26.92

HBV-ACLF-16	30	M	30.5	100	131	451	80	135	2.73	6.45	30.05
HBV-ACLF-17	54	M	29.5	41	71	283	113	140	1.55	NA	24.29
HBV-ACLF-18	57	M	34.1	633	432	381	69	137	2.42	5.81	28.06
HBV-ACLF-19	45	F	28.4	311	266	290	51	135	1.93	9.18	24.49
HBV-ACLF-20	43	M	27.2	372	157	278	51	140	2.72	4.53	28.18
HBV-ACLF-21	60	M	29.2	95	101	405	61	139	1.84	3.91	25.22
HBV-ACLF-22	34	M	26.3	894	704	275	51	139	1.64	6.93	22.47
HBV-ACLF-23	39	M	28.8	660	280	302	79	143	2.07	5.29	25.43
HBV-ACLF-24	46	M	36.6	302	224	512	122	138	2.67	3.72	33.35

Supplemental Table S3. List of the 2381 differentially expressed genes.

Gene ID	Gene short name	Locus
XLOC_000006	CICP27	1:89294-174387
XLOC_000031	ISG15	1:1001137-1014541
XLOC_000065	PRKCZ	1:2050425-2220738
XLOC_000067	SKI	1:2228616-2310262
XLOC_000070	PLCH2	1:2415550-2526616
XLOC_000112	VAMP3	1:7771268-7781434
XLOC_000145	NMNAT1,RP11-807G9.2	1:9922112-9989842
XLOC_000148	RBP7	1:9997205-10016020
XLOC_000155	PGD	1:10210521-10420144
XLOC_000170	UBIAD1	1:11273168-11299536
XLOC_000173	FBXO44	1:11648333-11663334
XLOC_000175	DRAXIN	1:11691728-11725857
XLOC_000185	TNFRSF8	1:12063376-12144233
XLOC_000186	MIR7846,TNFRSF1B	1:12166729-12211765
XLOC_000266	PADI4	1:17308160-17364027
XLOC_000292	OTUD3	1:19882393-19912945
XLOC_000302	CDA	1:20588947-20618908
XLOC_000314	NBPF3	1:21440080-21485005
XLOC_000315	ALPL	1:21509371-21578412
XLOC_000331	ZBTB40	1:22451839-22531157
XLOC_000363	RCAN3	1:24501300-24556024
XLOC_000367	CLIC4	1:24745356-24844324
XLOC_000368	-	1:24857274-24877676
XLOC_000379	LDLRAP1	1:25543579-25575359
XLOC_000381	MAN1C1	1:25616798-25788649
XLOC_000425	SYTL1	1:27341615-27367169
XLOC_000431	FGR	1:27610572-27636267
XLOC_000438	RNU6-1245P,STX12	1:27773182-27824795
XLOC_000444	THEMIS2	1:27872511-27914820
XLOC_000448	-	1:28143209-28146654
XLOC_000470	EPB41	1:28886974-29123935
XLOC_000489	SERINC2	1:31409564-31434733
XLOC_000505	LCK	1:32251238-32286167
XLOC_000550	AGO4	1:35807598-35857893
XLOC_000566	MIR6732,ZC3H12A	1:37454878-37484448
XLOC_000623	EXO5	1:40477204-40517940
XLOC_000652	PPIH,RP5-994D16.3	1:42658246-42681659
XLOC_000691	BTBD19,PLK3	1:44799735-44843063
XLOC_000715	FAAHP1	1:46423580-46446216
XLOC_000791	GPX7	1:52602371-52609294
XLOC_000803	PODN	1:53062051-53085807
XLOC_000830	C1orf177,DHCR24	1:54775925-54888850
XLOC_000908	LEPR,LEPROT	1:65420586-65641559
XLOC_000910	PDE4B	1:65792513-66374646
XLOC_000914	TCTEX1D1	1:66752213-66779320
XLOC_000920	IL12RB2	1:67307139-67402722
XLOC_000925	GADD45A	1:67685060-67688338
XLOC_000996	PTGFR	1:78229598-78539749

XLOC_001020	PRKACB	1:84076330-84238498
XLOC_001160	CDC14A	1:100351733-100520277
XLOC_001169	RP4-575N6.5,S1PR1	1:101228673-101271365
XLOC_001208	GPSM2	1:108815897-108963501
XLOC_001217	CELSR2	1:109249661-109275750
XLOC_001220	CYB561D1	1:109494046-109502932
XLOC_001223	GSTM1,GSTM2,GSTM4	1:109656044-109709551
XLOC_001304	SLC22A15	1:115976264-116072734
XLOC_001312	CD2	1:116754384-116769448
XLOC_001356	LINC00869	1:120911312-121052167
XLOC_001358	FCGR1B	1:121087198-121146826
XLOC_001379	FCGR1C,RP5-998N21.4	1:143874710-143934776
XLOC_001467	LINC00869,PPIAL4C,RP11-353N4.6	1:149558468-149747812
XLOC_001471	FCGR1A	1:149782661-149813681
XLOC_001521	SNX27	1:151611830-151699091
XLOC_001529	AL450992.2	1:151836991-151856355
XLOC_001569	S100A9	1:153357673-153361500
XLOC_001595	ATP8B2	1:154325534-154351307
XLOC_001597	IL6R	1:154402327-154469552
XLOC_001609	ZBTB7B	1:155002629-155062775
XLOC_001641	SEMA4A	1:156147365-156182765
XLOC_001661	RP11-71G12.1	1:157177231-157194419
XLOC_001663	-	1:157400269-157419422
XLOC_001675	CD1D	1:158164104-158186416
XLOC_001688	MNDA	1:158830915-158850176
XLOC_001694	FCER1A	1:159283854-159308224
XLOC_001706	FCRL6	1:159800326-159820258
XLOC_001725	SLAMF7	1:160739056-160758210
XLOC_001742	FCER1G,MIR5187,NDUFS2,TOMM40L	1:161166387-161238302
XLOC_001749	FCGR2A,FCGR2B,FCGR2C,HSPA6,HSPA7,RP11-25K21.6	1:161505265-161678933
XLOC_001756	ATF6	1:161766292-161975996
XLOC_001759	C1orf226,NOS1AP,RP11-565P22.6	1:162069773-162386818
XLOC_001765	UAP1	1:162561505-162600010
XLOC_001822	CD247	1:167430530-167518610
XLOC_001840	XCL1	1:168576099-168582077
XLOC_001884	FASLG	1:172658990-172670744
XLOC_001956	QSOX1	1:180154808-180204030
XLOC_001985	NPL	1:182789292-182887847
XLOC_001987	LAMC1	1:183023353-183145592
XLOC_001992	RGL1	1:183636084-183928531
XLOC_001998	C1orf21	1:184387007-184639044
XLOC_002021	PLA2G4A	1:186828952-186989026
XLOC_002042	RGS18	1:192158362-192390374
XLOC_002046	RGS1	1:192575726-192580031
XLOC_002047	RGS2	1:192590119-192812419
XLOC_002099	GPR25	1:200872917-200874930
XLOC_002100	C1orf106	1:200891047-200915735
XLOC_002128	LGR6	1:202146925-202321648

XLOC_002140	PPFIA4		1:203026497-203078740
XLOC_002144	BTG2		1:203305419-203314770
XLOC_002147	ATP2B4		1:203626560-203744209
XLOC_002149	LAX1		1:203765128-203778286
XLOC_002153	SOX13		1:204073105-204127743
XLOC_002186	SRGAP2		1:206186178-206464443
XLOC_002201	PFKFB2		1:207006844-207099993
XLOC_002206	CD55		1:207321408-207375427
XLOC_002209	CR1		1:207496146-207641981
XLOC_002223	G0S2		1:209661363-209734950
XLOC_002226	TRAF3IP3		1:209756014-209784665
XLOC_002256	ATF3		1:212565333-212621656
XLOC_002305		1-Mar	1:220786697-220820577
XLOC_002308	HLX		1:220832737-220885225
XLOC_002329	DEGS1		1:224175755-224199330
XLOC_002347	H3F3A		1:226053757-226072001
XLOC_002373	ARF1,MIR3620		1:228082659-228099216
XLOC_002380	OBSCN		1:228164085-228378876
XLOC_002388	FTH1P2		1:228687414-228687826
XLOC_002389	RHOU		1:228735076-228746669
XLOC_002535	CNST		1:246566443-246668584
XLOC_002539	SCCPDH		1:246723926-246768311
XLOC_002552	NLRP3		1:247415874-247747062
XLOC_002600	LYPD8		1:248718648-248808520
XLOC_002635	HES4		1:998884-1000172
XLOC_002723	GPR153		1:6247102-6260975
XLOC_002729	PLEKHG5,TNFRSF25		1:6424787-6520061
XLOC_002763	SLC2A5		1:9035106-9094228
XLOC_002810	DHRS3		1:12567909-12618274
XLOC_002866	ARHGEF19		1:16197853-16212609
XLOC_002892	PADI2		1:17066760-17119435
XLOC_002894	PADI4		1:17308160-17364027
XLOC_002898	ALDH4A1,IFFO2,RP13-279N23.2,TAS1R2		1:18839598-18956686
XLOC_002930	ECE1		1:21217164-21345504
XLOC_002932	NBPF2P		1:21423112-21427967
XLOC_002958	TCEA3		1:23380935-23424769
XLOC_002960	E2F2		1:23506429-23531336
XLOC_002983	RUNX3		1:24899266-24965121
XLOC_002997	-		1:25597317-25601523
XLOC_003004	PAFAH2		1:25959632-25998157
XLOC_003011	ZNF683		1:26361633-26374522
XLOC_003038	AHDC1		1:27522543-27604431
XLOC_003039	FGR		1:27610572-27636267
XLOC_003081	LAPTM5		1:30732422-30757820
XLOC_003084	SDC3		1:30869466-30908761
XLOC_003112	LCK		1:32251238-32286167
XLOC_003126	RNF19B		1:32928313-32965070
XLOC_003130	A3GALT2,PHC2		1:33306765-33431075
XLOC_003153	CLSPN		1:35720217-35769967

XLOC_003160	COL8A2	1:36095235-36125708
XLOC_003168	CSF3R,MRPS15	1:36455717-36483316
XLOC_003183	MTF1,YRDC	1:37793801-37859625
XLOC_003203	HPCAL4	1:39678179-39691689
XLOC_003225	RIMS3	1:40620678-40665705
XLOC_003227	CITED4	1:40861050-40862366
XLOC_003233	-	1:41302917-41305970
XLOC_003251	SLC2A1	1:42925380-42958914
XLOC_003335	SPATA6	1:48295371-48472223
XLOC_003365	ORC1	1:52372637-52404459
XLOC_003372	ECHDC2	1:52895909-53051703
XLOC_003379	SLC1A7	1:53087178-53142632
XLOC_003399	SSBP3	1:54133073-54413479
XLOC_003404	TTC22	1:54775925-54888850
XLOC_003433	JUN	1:58780079-58784982
XLOC_003480	JAK1	1:64745094-64966603
XLOC_003606	GNG5	1:84479258-84575307
XLOC_003607	CTBS	1:84479258-84575307
XLOC_003625	MIR7856,ODF2L	1:86346662-86396342
XLOC_003649	GBP5	1:89256194-89274237
XLOC_003666	TGFBR3	1:91680342-91906335
XLOC_003677	EVI5	1:92508695-92792404
XLOC_003707	ARHGAP29	1:94148987-94275068
XLOC_003729	RPL7P9	1:96678873-96679620
XLOC_003735	DPYD	1:97077742-97921049
XLOC_003785	SLC25A24	1:108128493-108202743
XLOC_003803	PSRC1	1:109279555-109283186
XLOC_003805	SORT1	1:109309319-109397976
XLOC_003807	AMIGO1	1:109503935-109509748
XLOC_003829	KCNA3	1:110653559-110692458
XLOC_003856	PPM1J,RHOC,RP11-426L16.10	1:112673140-112715477
XLOC_003867	BCL2L15	1:113761741-113914086
XLOC_003900	CD2	1:116754384-116769448
XLOC_003901	FTH1P22	1:116772953-116806447
XLOC_003935	ZNF697	1:119618979-119648246
XLOC_003940	NOTCH2,RP5-1042I8.7	1:119909254-120070143
XLOC_004075	OTUD7B	1:149937811-150010744
XLOC_004091	CTSS	1:150724759-150776403
XLOC_004118	LINGO4,RORC	1:151800263-151831872
XLOC_004121	THEM4	1:151870473-151909838
XLOC_004124	S100A11	1:151994530-152047907
XLOC_004139	IVL	1:152897539-152913104
XLOC_004152	S100A12	1:153373705-153375649
XLOC_004154	S100A8	1:153390031-153391188
XLOC_004160	S100A6	1:153531100-153536608
XLOC_004181	RAB13	1:153981616-153986375
XLOC_004202	PBXIP1	1:154944062-154956526
XLOC_004211	GBA	1:155230974-155244699
XLOC_004225	RIT1	1:155897807-155911968
XLOC_004242	IQGAP3	1:156525404-156572604

XLOC_004257	ARHGEF11	1:156934839-157045877
XLOC_004265	FCRL5	1:157512438-157552523
XLOC_004269	FCRL3	1:157673990-157700964
XLOC_004309	FCRL6	1:159800326-159820258
XLOC_004319	IGSF8	1:160091339-160098943
XLOC_004326	SLAMF6	1:160485029-160523370
XLOC_004328	SLAMF1	1:160608099-160647328
XLOC_004363	SH2D1B	1:162394698-162412145
XLOC_004393	GPA33	1:167049624-167090642
XLOC_004397	CD247	1:167430530-167518610
XLOC_004400	CREG1	1:167528867-167553767
XLOC_004413	XCL2	1:168540764-168544299
XLOC_004423	F5	1:169511804-169586588
XLOC_004429	KIFAP3	1:169921260-170085208
XLOC_004500	RP11-568K15.1,RP4-798P15.3,SEC16B	1:177923955-178038007
XLOC_004545	GLUL	1:182379109-182392316
XLOC_004564	NCF2	1:183460873-183598246
XLOC_004566	ARPC5	1:183620845-183635977
XLOC_004571	COLGALT2	1:183929853-184037753
XLOC_004574	EDEM3	1:184690230-184754913
XLOC_004594	PTGS2	1:186671790-186681446
XLOC_004646	-	1:198473268-198519872
XLOC_004666	KIF21B	1:200966955-201023741
XLOC_004682	ARL8A	1:202122857-202144743
XLOC_004697	ADIPOR1	1:202940635-202958572
XLOC_004705	CHI3L1	1:203178611-203186768
XLOC_004706	CHIT1	1:203212826-203274841
XLOC_004721	ETNK2	1:204131061-204152003
XLOC_004746	RAB29	1:205766489-205775713
XLOC_004747	SLC41A1	1:205788911-205813862
XLOC_004765	IL10	1:206767601-206772494
XLOC_004766	FAIM3	1:206885139-206923247
XLOC_004785	PLXNA2	1:208022241-208244320
XLOC_004810	SLC30A1	1:211571567-211578742
XLOC_004818	LPGAT1	1:211743450-211853703
XLOC_004898	TLR5	1:223105313-223143304
XLOC_004918	LBR	1:225401494-225428925
XLOC_004922	RP11-285F7.2,TMEM63A	1:225810091-225883467
XLOC_004932	PARP1	1:226360690-226408075
XLOC_004935	ITPKB	1:226631624-226739358
XLOC_004992	-	1:228929960-228949736
XLOC_005033	PCNXL2,RP5-862P8.3	1:232950577-233295762
XLOC_005061	LYST	1:235660946-235883640
XLOC_005083	CHRM3-AS2	1:239386564-239916955
XLOC_005115	CEP170	1:243124427-243851925
XLOC_005190	ZNF692	1:248850005-248864796
XLOC_005255	-	1:84579326-84582446
XLOC_005281	-	1:108088837-108090942
XLOC_005282	-	1:108091165-108100971

XLOC_005465	ZMYND11	10:134464-254637
XLOC_005504	AKR1C1,AKR1C3,RP11-499O7.4	10:4922563-5135226
XLOC_005522	PFKFB3	10:6144895-6335982
XLOC_005527	PRKCQ-AS1,RP11-554I8.1	10:6426923-6842906
XLOC_005538	GATA3	10:8053603-8075207
XLOC_005559	ECHDC3	10:11742365-11764070
XLOC_005570	OPTN	10:12896624-13140333
XLOC_005600	VIM	10:17210905-17237835
XLOC_005619	PLXDC2	10:19816025-20289856
XLOC_005652	MSRB2	10:23095505-23122013
XLOC_005659	OTUD1	10:23438842-23444691
XLOC_005674	APBB1IP	10:26437896-26571485
XLOC_005712	MAP3K8	10:30433891-30461833
XLOC_005713	HNRNPA1P32	10:30509127-30537005
XLOC_005720	ZEB1	10:31187882-31529814
XLOC_005808	CSGALNACT2	10:43136823-43185308
XLOC_005839	ALOX5	10:45373889-45453121
XLOC_005847	-	10:46091013-46101937
XLOC_005902	ASAH2B	10:50739317-50885675
XLOC_005929	UBE2D1	10:58334974-58370753
XLOC_005942	CDK1	10:60778330-60794852
XLOC_005950	ZNF365	10:62374191-62672011
XLOC_006005	-	10:69047678-69072140
XLOC_006006	SRGN	10:69080533-69105263
XLOC_006010	HKDC1	10:69215332-69268148
XLOC_006012	TSPAN15	10:69451472-69507753
XLOC_006015	C10orf35	10:69630250-69633890
XLOC_006018	COL13A1	10:69801101-69964275
XLOC_006039	DDIT4	10:72273919-72276036
XLOC_006046	MCU	10:72692130-72887757
XLOC_006074	PLAU	10:73909176-73922777
XLOC_006077	ADK,MRPL35P3	10:74151184-74710090
XLOC_006081	KAT6B	10:74825143-75032622
XLOC_006082	SAMD8	10:75094431-75182123
XLOC_006089	C10orf11	10:75430570-76560994
XLOC_006116	PPIF	10:79347084-79576977
XLOC_006155	CDHR1	10:84194557-84219621
XLOC_006158	CCSER2	10:84328573-84518521
XLOC_006197	LIPN	10:88759976-88783646
XLOC_006245	CEP55	10:93496631-93529092
XLOC_006255	TBC1D12	10:94402250-94613905
XLOC_006265	C10orf131,CC2D2B,ENTPD1,RP11-248J23.6,RP11-248J23.7,RP11-429G19.3,RP11-690P14.4	10:95711778-96090238
XLOC_006281	FRAT1	10:97319266-97321915
XLOC_006294	GOLGA7B	10:97828477-98030828
XLOC_006312	SCD	10:100347101-100364834
XLOC_006339	ELOVL3	10:102226301-102229589
XLOC_006370	GSTO1,GSTO2	10:104235355-104304945
XLOC_006406	SHOC2	10:110919546-111013944

XLOC_006451	ENO4	10:116828760-117126586
XLOC_006469	GRK5	10:119207588-119459880
XLOC_006497	PLEKHA1	10:122374526-122442602
XLOC_006541	PTPRE,RP11-4C20.4	10:127907055-128085889
XLOC_006572	VENTX	10:133232153-133241953
XLOC_006575	CYP2E1,MTG1,PAOX,RP11-108K14.8,SCART1	10:133379233-133569835
XLOC_006615	DIP2C	10:273342-689743
XLOC_006659	GDI2	10:5684754-5842132
XLOC_006669	PRKCQ	10:6426923-6842906
XLOC_006671	SFMBT2	10:7158478-7411486
XLOC_006738	NMT2	10:15095384-15168717
XLOC_006741	FAM171A1	10:15211642-15371062
XLOC_006751	ST8SIA6	10:17315199-17455486
XLOC_006830	MPP7	10:28050992-28335264
XLOC_006858	SVILP1	10:30671565-30717266
XLOC_006869	KIF5B	10:32008876-32056439
XLOC_006906	NAMPTP1	10:36521720-36524234
XLOC_006912	RP11-258F22.1,ZNF248	10:37775370-37860791
XLOC_006981	NCOA4	10:45825476-46030714
XLOC_006983	ANTXRLP1	10:46196813-46273557
XLOC_007024	C10orf128	10:49154409-49188585
XLOC_007034	SGMS1	10:50305585-50647252
XLOC_007065	IPMK	10:58191327-58267958
XLOC_007074	CCDC6	10:59788762-59907020
XLOC_007076	ANK3	10:60026297-60733705
XLOC_007083	RTKN2	10:62183034-62268707
XLOC_007086	EGR2	10:62811995-62919900
XLOC_007139	AIFM2,TYSND1	10:70052795-70146847
XLOC_007148	PRF1	10:70478820-70881173
XLOC_007156	PSAP	10:71396933-71851369
XLOC_007160	SPOCK2	10:72059013-72089032
XLOC_007166	MICU1	10:72367326-72626191
XLOC_007211	ZNF503	10:75269818-75411842
XLOC_007228	DLG5	10:77782865-77926801
XLOC_007320	ANKRD22	10:88802729-88851947
XLOC_007349	CPEB3	10:92046691-92291087
XLOC_007363	MYOF	10:93306428-93482476
XLOC_007385	ALDH18A1	10:95605928-95656833
XLOC_007395	PIK3AP1	10:96593311-96720514
XLOC_007401	FRAT2	10:97325426-97343203
XLOC_007432	DNMBP	10:99875576-100009967
XLOC_007436	CHUK,ERLIN1	10:100150093-100267689
XLOC_007462	FBXW4	10:101610663-101695491
XLOC_007487	NT5C2	10:102918143-103197627
XLOC_007499	COL17A1,MIR936	10:104031011-104086002
XLOC_007503	ITPRIP	10:104309546-104338467
XLOC_007547	ABLIM1	10:114430957-114801441
XLOC_007558	ENO4,KIAA1598	10:116828760-117126586
XLOC_007582	PRDX3	10:119167702-119178833

XLOC_007617	CHST15	10:123706206-124093607
XLOC_007652	MKI67	10:128096604-128126391
XLOC_007736	-	10:23447559-23449396
XLOC_007763	-	10:46283963-46284443
XLOC_007788	-	10:92593082-92695665
XLOC_007825	-	10:124093939-124097076
XLOC_007850	ATHL1,RP11-326C3.2	11:287037-297974
XLOC_007851	IFITM1,IFITM2,RP11-326C3.10,RP11-326C3.11,RP11-326C3.12,RP11-326C3.15	11:307630-344670
XLOC_007855	SIGIRR	11:405714-442296
XLOC_007863	RASSF7,RP11-496I9.1	11:532068-564021
XLOC_007869	TALDO1	11:747328-785391
XLOC_007897	AC051649.12,LSP1,hsa-mir-7847	11:1851235-1916360
XLOC_007899	TNNT3	11:1916861-1938706
XLOC_007909	CD81	11:2327667-2397691
XLOC_008005	OR52L2P	11:6040167-6065138
XLOC_008019	GVINP1	11:6691591-6746511
XLOC_008064	SWAP70	11:9664076-9753063
XLOC_008069	ADM	11:10304679-10307397
XLOC_008070	AMPD3	11:10308312-10541247
XLOC_008102	FAR1	11:13668526-13732349
XLOC_008146	LDHA	11:18394387-18408425
XLOC_008238	PRRG4	11:32829739-32858123
XLOC_008257	CAT	11:34438924-34472064
XLOC_008264	CD44	11:35138682-35530300
XLOC_008272	LDLRAD3	11:35943980-36232138
XLOC_008278	PRR5L	11:36296287-36510537
XLOC_008324	CD82	11:44564426-44622071
XLOC_008360	SPI1	11:47354743-47417460
XLOC_008371	PTPRJ	11:47980486-48170957
XLOC_008545	OR10Y1P,STX3	11:59712822-59810975
XLOC_008552	MS4A3	11:60056558-60080853
XLOC_008556	MS4A4A	11:60280540-60308972
XLOC_008557	MS4A14,MS4A6E,MS4A7	11:60334830-60418135
XLOC_008572	CD6	11:60971372-61020377
XLOC_008574	CD5	11:61102394-61128735
XLOC_008624	LGALS12	11:63506083-63516774
XLOC_008625	RARRES3	11:63536808-63546462
XLOC_008629	RTN3	11:63681445-63768775
XLOC_008632	NAA40	11:63938709-63958479
XLOC_008671	SLC22A20	11:65213839-65242757
XLOC_008696	CTSW	11:65879804-65888585
XLOC_008731	RAD9A,TBC1D10C	11:67317413-67425649
XLOC_008751	ALDH3B1	11:68008577-68030461
XLOC_008810	FOLR3	11:72114868-72141418
XLOC_008816	INPPL1	11:72223304-72245664
XLOC_008838	P2RY2,RP11-800A3.4	11:73216100-73249867
XLOC_008842	RELT,RP11-809N8.4	11:73376263-73598505
XLOC_008847	PLEKHB1	11:73646177-73662872

XLOC_008874	RPS3,SNORD15A	11:75399485-75422280
XLOC_008877	SERPINH1	11:75562055-75572783
XLOC_008882	DGAT2	11:75758454-75803415
XLOC_008895	ACER3	11:76860866-77027086
XLOC_008898	CAPN5	11:77066931-77126155
XLOC_008900	MYO7A	11:77128263-77215785
XLOC_008927	RPS28P7	11:81879850-82718082
XLOC_008952	-	11:85952028-85956624
XLOC_008963	PRSS23	11:86791058-86955391
XLOC_009034	SLC36A4	11:93100625-93198037
XLOC_009054	FUT4,PIWIL4,RP11-867G2.4	11:94539206-94925521
XLOC_009063	RP11-60C6.5,SRSF8	11:95037332-95071745
XLOC_009067	ENDOD1	11:95089799-95134427
XLOC_009126	RAB39A	11:107928437-107968144
XLOC_009137	ZC3H12C	11:110092951-110171872
XLOC_009166	NCAM1	11:112959278-113314437
XLOC_009180	ZBTB16	11:114059578-114356571
XLOC_009204	AP000892.6,PAFAH1B2,SIDT2,TAGLN	11:117144266-117413268
XLOC_009210	RP11-728F11.4	11:117427772-117877486
XLOC_009215	-	11:118225433-118226383
XLOC_009217	CD3E	11:118304433-118316175
XLOC_009218	CD3G	11:118334582-118355161
XLOC_009222	TMEM25,TTC36	11:118435722-118603033
XLOC_009300	VWA5A	11:124115361-124147721
XLOC_009321	ROBO3	11:124865385-124881471
XLOC_009326	SLC37A2	11:125062920-125111855
XLOC_009380	APLP2	11:130069836-130144811
XLOC_009381	ST14	11:130159561-130210376
XLOC_009408	GLB1L2	11:134331873-134412078
XLOC_009427	ANO9,SIGIRR	11:405714-442296
XLOC_009456	CTSD,IFITM10,KRTAP5-4,RP11-295K3.1	11:1604267-1764025
XLOC_009481	CDKN1C	11:2883212-2885881
XLOC_009489	OSBPL5	11:3087019-3166739
XLOC_009506	RHOG	11:3665586-3840983
XLOC_009592	PRKCDBP	11:6318945-6320647
XLOC_009593	APBB1	11:6390381-6419414
XLOC_009598	DCHS1	11:6621261-6655854
XLOC_009609	CYB5R2	11:7665099-7677222
XLOC_009638	DENND5A	11:9138537-9265526
XLOC_009649	RP11-1H15.1,SBF2	11:9754769-10294215
XLOC_009652	RP11-351I24.1	11:10300474-10303822
XLOC_009654	MTRNR2L8,RNF141	11:10308312-10541247
XLOC_009656	MRVI1	11:10541271-10693988
XLOC_009670	DKK3	11:11963105-12009769
XLOC_009691	RP11-21L19.1,RRAS2	11:13962688-14364506
XLOC_009742	E2F8	11:19182029-19281426
XLOC_009824	LMO2	11:33858575-33892289
XLOC_009880	TP53I11	11:44701526-44951306

XLOC_009922	MYBPC3	11:47331396-47352702
XLOC_009923	RP11-750H9.5,SPI1	11:47354743-47417460
XLOC_010031	PRG2,RP11-872D17.8,SLC43A3	11:57386793-57427580
XLOC_010033	SLC43A1	11:57484439-57515788
XLOC_010082	MPEG1	11:59169996-59213523
XLOC_010101	TCN1	11:59852799-59866575
XLOC_010102	AP001257.1,MS4A6A	11:60153856-60184666
XLOC_010103	MIR6503,MS4A4E	11:60199286-60243152
XLOC_010132	FTH1	11:61949820-61969490
XLOC_010193	MEN1	11:64803509-64811294
XLOC_010194	CDC42BPG	11:64823118-64844850
XLOC_010221	LTBP3	11:65525021-65558930
XLOC_010226	AP5B1	11:65721198-65781033
XLOC_010232	FOSL1	11:65890105-65900573
XLOC_010245	CD248	11:66312852-66319237
XLOC_010249	SLC29A2	11:66347949-66372463
XLOC_010255	CTSF	11:66563462-66568841
XLOC_010278	CORO1B,PTPRCAP	11:67427989-67448783
XLOC_010294	UNC93B1,UNC93B5	11:67711701-68007037
XLOC_010314	CPT1A	11:68754619-68844410
XLOC_010397	KCNE3	11:74330314-74486051
XLOC_010448	PAK1	11:77321706-77477030
XLOC_010464	GAB2	11:78139770-78574874
XLOC_010485	PRCP	11:82822696-83071923
XLOC_010506	SYTL2	11:85694223-85811175
XLOC_010509	PICALM	11:85957683-86069882
XLOC_010515	ME3	11:86431589-86672636
XLOC_010582	SLC36A4	11:93100625-93198037
XLOC_010587	SMCO4	11:93478471-93543508
XLOC_010595	RP11-680H20.1	11:94188448-94188997
XLOC_010641	MMP8	11:102711727-102727050
XLOC_010659	PDGFD	11:103675993-104164379
XLOC_010663	RP11-693N9.2	11:104901548-104919073
XLOC_010666	CARD16,CARD17,CASP1	11:105023530-105101445
XLOC_010700	KDELC2	11:108472104-108498631
XLOC_010759	USP28	11:113797873-113875627
XLOC_010790	RP11-109L13.1	11:117134999-117138858
XLOC_010799	FXYD2,FXYD6,FXYD6-FXYD2	11:117427772-117877486
XLOC_010809	CD3D	11:118334582-118355161
XLOC_010820	BCL9L	11:118882614-118958559
XLOC_010825	HYOU1	11:119044187-119057252
XLOC_010868	HSPA8,SNORD14C,SNORD14D	11:123057488-123228277
XLOC_010920	FEZ1	11:125445744-125499528
XLOC_010945	-	11:128290053-128294076
XLOC_010946	ETS1	11:128458760-128587558
XLOC_011002	B3GAT1	11:134331873-134412078
XLOC_011017	-	11:10296360-10300423
XLOC_011055	-	11:65522015-65524859
XLOC_011137	CCND2	12:4248764-4305350
XLOC_011155	LTBR,RP1-102E24.8	12:6346842-6413803

XLOC_011161	GAPDH	12:6533926-6538374
XLOC_011170	LAG3	12:6772363-6778456
XLOC_011177	C12orf57,RNU7-1	12:6942977-6946029
XLOC_011205	CLEC4A,FAM66C,ZNF705A	12:8123631-8221115
XLOC_011213	CLEC6A	12:8455925-8481063
XLOC_011216	CLEC4D	12:8504997-8568035
XLOC_011229	A2MP1,KRT17P8,LINC00987,RP11-118B22.4,RP11-436I9.6	12:9055585-9275817
XLOC_011235	RP11-726G1.1	12:9467551-9584141
XLOC_011237	CLEC2D,GOT2P3,RP11-705C15.2,RP11-705C15.3,RP11-705C15.5,RP11-75L1.1	12:9633418-9740556
XLOC_011238	KLRF1	12:9827309-9870830
XLOC_011246	CLEC7A	12:10116290-10130261
XLOC_011247	TMEM52B	12:10158300-10191801
XLOC_011249	KLRD1	12:10226057-10330519
XLOC_011250	RP11-277P12.20	12:10356794-10523135
XLOC_011252	RP11-291B21.2	12:10553336-10579451
XLOC_011265	ETV6	12:11649494-11895503
XLOC_011288	HTR7P1,RP11-377D9.3	12:13000419-13040679
XLOC_011310	PTPRO	12:15107782-15598592
XLOC_011316	MGST1	12:16347141-16610594
XLOC_011335	PLEKHA5	12:19129690-19376550
XLOC_011385	SSPN	12:26120025-26833287
XLOC_011412	FAR2	12:29148873-29381209
XLOC_011447	FGD4	12:32399528-32646050
XLOC_011470	LRRK2	12:40186008-40369285
XLOC_011604	METTL7A	12:50923471-50932521
XLOC_011625	ACVRL1	12:51906907-51923361
XLOC_011626	ACVR1B	12:51951666-51997450
XLOC_011628	GRASP	12:52006928-52015966
XLOC_011629	NR4A1	12:52022831-52059507
XLOC_011654	SOAT2	12:53102958-53124538
XLOC_011661	ESPL1	12:53268287-53293643
XLOC_011663	SP1	12:53380036-53416453
XLOC_011736	CDK2,DGKA	12:55901412-55973317
XLOC_011746	COQ10A	12:56266748-56271119
XLOC_011760	LRP1	12:57088893-57213361
XLOC_011771	DTX3	12:57604621-57609804
XLOC_011772	ARHGEF25	12:57610179-57633355
XLOC_011839	MSRB3	12:65278552-65642372
XLOC_011851	IRAK3,MIR6502	12:66188802-66257434
XLOC_011858	DYRK2	12:67648337-67729475
XLOC_011877	LYZ	12:69348340-69354734
XLOC_011884	-	12:69636558-69636668
XLOC_011911	ATXN7L3B	12:74537826-74545430
XLOC_011918	GLIPR1	12:75480322-75511636
XLOC_011970	TMTC2	12:82686874-83134870
XLOC_012040	SOCS2	12:93542462-93583487
XLOC_012045	PLXNC1	12:94148722-94459988

XLOC_012080	APAF1	12:98613404-99984654
XLOC_012106	DRAM1	12:101877348-102062149
XLOC_012137	KIAA1033	12:105107323-105412817
XLOC_012149	TMEM263	12:106953477-106987195
XLOC_012172	UNG	12:109097573-109110992
XLOC_012201	SH2B3	12:111405947-111451963
XLOC_012205	ACAD10,ALDH2,RP11-162P23.2	12:111686050-111814448
XLOC_012220	RPH3A	12:112570379-112898881
XLOC_012233	SDSL	12:113392444-113438277
XLOC_012298	CCDC64	12:119988885-120094797
XLOC_012326	P2RX7,RP11-340F14.6	12:121132796-121191518
XLOC_012329	CAMKK2	12:121237361-121298308
XLOC_012335	ORAI1	12:121626387-121643151
XLOC_012374	TCTN2	12:123671112-123708403
XLOC_012432	GLT1D1	12:128843300-128985103
XLOC_012524	FBXL14	12:1523100-1647243
XLOC_012574	TNFRSF1A	12:6328756-6342114
XLOC_012586	LPAR5	12:6614697-6636447
XLOC_012599	C1R,C1RL	12:7080208-7158945
XLOC_012603	CD163,CD163L1	12:7346684-7503893
XLOC_012608	CLEC4C	12:7724363-7751605
XLOC_012614	SLC2A3	12:7919169-7936275
XLOC_012617	C3AR1	12:8058301-8066471
XLOC_012633	CLEC4E,RP11-561P12.5	12:8504997-8568035
XLOC_012651	KLRB1	12:9594550-9607886
XLOC_012664	CLEC7A	12:10116290-10130261
XLOC_012665	OLR1	12:10158300-10191801
XLOC_012668	KLRC1,KLRC2,KLRC3,KLRC4,KLRC4-KLRK1,KLRK1,NKG2-E,RP11-277P12.9	12:10356794-10523135
XLOC_012671	STYK1	12:10618938-10674318
XLOC_012672	YBX3	12:10699088-10725972
XLOC_012702	MANSC1	12:12326055-12350541
XLOC_012724	PLBD1	12:14356841-14771167
XLOC_012765	LDHB	12:21635341-21775581
XLOC_012780	BCAT1	12:24810021-24960158
XLOC_012786	KRAS	12:25108419-25326458
XLOC_012833	TMTC1	12:29500839-29784759
XLOC_012877	PKP2	12:32790744-32896840
XLOC_012900	CPNE8	12:38646821-38909592
XLOC_012905	ABCD2,AC121334.1,KIF21A	12:39293227-39620414
XLOC_012950	NELL2	12:44508274-44921848
XLOC_012963	SLC38A1	12:46182830-46275957
XLOC_012972	PCED1B-AS1	12:47075706-47279021
XLOC_012985	VDR	12:47840614-47943048
XLOC_013018	WNT10B	12:48965339-48982587
XLOC_013021	RHEBL1	12:49064684-49070025
XLOC_013036	NCKAP5L	12:49790676-49843129
XLOC_013044	LIMA1	12:50175787-50283546
XLOC_013099	KRT72,KRT73	12:52585588-52630855

XLOC_013119	ITGB7	12:53157662-53209759
XLOC_013144	GPR84	12:54353660-54588659
XLOC_013153	TESPA1	12:54915350-54985215
XLOC_013167	CD63	12:55681545-55731249
XLOC_013224	AGAP2	12:57723361-57760407
XLOC_013287	GNS	12:64713324-64759447
XLOC_013303	AC078889.1,RBMS1P1,RP11-335I12.2	12:66188802-66257434
XLOC_013323	RP11-81H14.1,RP11-81H14.2	12:68332887-68451932
XLOC_013425	LIN7A	12:80792365-81759553
XLOC_013458	DUSP6	12:89347231-89360316
XLOC_013484	RP11-887P2.3	12:93640821-93641586
XLOC_013508	NTN4	12:95649254-95791152
XLOC_013513	LTA4H	12:95972505-96110323
XLOC_013534	SLC9A7P1	12:98450442-98457293
XLOC_013545	RP11-135F9.3,UHRF1BP1L	12:100028454-100142922
XLOC_013585	NT5DC3,RP11-341G23.4	12:103587272-103841236
XLOC_013602	CKAP4	12:106237876-106349332
XLOC_013610	MTERF2	12:106953477-106987195
XLOC_013611	CRY1	12:106991299-107123314
XLOC_013622	CMKLR1	12:108284832-108339559
XLOC_013627	CORO1C	12:108617707-108731933
XLOC_013644	TRPV4	12:109782935-109833436
XLOC_013645	GLTP	12:109850938-109880488
XLOC_013653	ANAPC7,ARPC3,RP11-478C19.2	12:110372899-110450422
XLOC_013659	HVCN1	12:110614026-110704950
XLOC_013723	TESC	12:117038922-117099479
XLOC_013729	WSB2	12:118013587-118062430
XLOC_013748	GCN1L1	12:120127025-120194721
XLOC_013781	CAMKK2	12:121237361-121298308
XLOC_013794	CLIP1	12:122271431-122422680
XLOC_013815	RILPL2	12:123383772-123438358
XLOC_013853	LINC00944	12:126703391-126772450
XLOC_013915	RNU6-1017P	12:131808975-131827634
XLOC_013929	-	12:132427401-132447244
XLOC_014160	C1QTNF9,RP11-307N16.6,SPATA13	13:23979699-24322535
XLOC_014174	PABPC3	13:25095867-25099254
XLOC_014179	NUPL1	13:25300123-25349800
XLOC_014229	ALOX5AP	13:30713477-30764426
XLOC_014247	KL	13:33016432-33066204
XLOC_014318	LACC1	13:43823908-43893932
XLOC_014377	ARL11	13:49628298-49642426
XLOC_014414	HNRNPA1L2,MRPS31P4,RP11-78J21.7	13:52600041-52644600
XLOC_014418	OLFM4	13:53008040-53052058
XLOC_014501	KLF5	13:73054975-73077542
XLOC_014508	LINC00381,LINC00402,RPL21P108	13:74231381-74444637
XLOC_014518	AC000403.4	13:76887550-76891135
XLOC_014593	DNAJC3	13:95677138-95794989

XLOC_014625	TM9SF2	13:99498535-99564127
XLOC_014662	RP11-153I24.4,TNFSF13B	13:108244081-108308505
XLOC_014670	IRS2	13:109751874-109808252
XLOC_014702	ATP11A	13:112686717-112887216
XLOC_014705	MCF2L	13:112894377-113099739
XLOC_014721	TMCO3	13:113455402-113554590
XLOC_014732	-	13:114154580-114179593
XLOC_014803	ZDHHC20	13:21372487-21459397
XLOC_014825	SACS	13:23328697-23433728
XLOC_014846	MTMR6	13:25228553-25288009
XLOC_014876	FLT3	13:28003267-28100732
XLOC_014888	SLC7A1	13:29509409-29595746
XLOC_015049	LCP1	13:46125687-46211871
XLOC_015051	KIAA0226L,LINC00563,PPP1R2P4	13:46211942-46438190
XLOC_015091	DLEU2,MIR15A,MIR3613	13:49954415-50849905
XLOC_015167	PCDH9	13:66302833-67230445
XLOC_015181	DACH1	13:71437965-71867192
XLOC_015195	KLF12	13:73686031-74134346
XLOC_015201	TBC1D4	13:75284664-75482659
XLOC_015208	AC000403.1,KCTD12	13:76880165-76886400
XLOC_015309	DOCK9	13:98793486-99088625
XLOC_015316	CCR12P	13:99393668-99417681
XLOC_015377	IRS2	13:109751874-109808252
XLOC_015386	RAB20	13:110523065-110561772
XLOC_015388	CARS2	13:110613081-110723339
XLOC_015419	LINC00565,RASA3	13:113926427-114132857
XLOC_015429	-	13:18746024-18746429
XLOC_015448	-	13:49642633-49645308
XLOC_015449	-	13:49645451-49646561
XLOC_015556	RP11-203M5.6	14:20427236-20443793
XLOC_015563	ANG,RNASE4	14:20684099-20707120
XLOC_015566	RNASE6,RP11-219E7.1	14:20781050-20787625
XLOC_015567	RNASE3	14:20891398-20892348
XLOC_015568	RP11-84C10.2	14:20897984-20936255
XLOC_015569	RNASE2	14:20955451-20956440
XLOC_015576	ARHGEF40	14:21016762-21104722
XLOC_015587	TRAV1-1	14:21612717-21632133
XLOC_015601	TRAV8-2,TRAV8-3	14:21846464-21866608
XLOC_015602	TRAV13-1	14:21868693-21869714
XLOC_015603	TRAV12-2	14:21887856-21888502
XLOC_015605	TRAV13-2	14:21918016-21920018
XLOC_015607	TRAV9-2	14:21941127-21941657
XLOC_015609	TRAV12-3	14:21965347-21966218
XLOC_015611	TRAV16	14:21990495-21990938
XLOC_015612	TRAV17	14:21997538-21998168
XLOC_015614	TRAV19	14:22007511-22008181
XLOC_015621	TRDV1	14:22096031-22096619
XLOC_015622	TRAV24	14:22105342-22105846
XLOC_015623	TRAV25	14:22112346-22113031
XLOC_015624	TRAV26-1	14:22123317-22124285

XLOC_015627	TRAV27	14:22147994-22148633
XLOC_015629	TRAV29DV5	14:22163237-22163870
XLOC_015640	AE000661.37,TRAC,TRAJ21,TRAJ23,TRAJ29,TRAJ32,TRAJ39,TRAJ54,TRAV38-2DV8,TRDC,TRDD3,TRDJ1,TRDJ2,TRDJ3,TRDJ4,TRDV2	14:22281104-22552155
XLOC_015745	RP11-104E19.1	14:24595722-24657774
XLOC_015963	LGALS3	14:55124109-55145413
XLOC_015978	PELI2	14:56117008-56301663
XLOC_016032	FLJ22447,PRKCH,RP11-47I22.4	14:61187558-61658696
XLOC_016033	HIF1A,RP11-618G20.1,SNAPC1	14:61681040-61796552
XLOC_016053	SYNE2	14:63852524-64338599
XLOC_016061	PLEKHG3	14:64703672-64879883
XLOC_016125	SIPA1L1	14:71292728-71741229
XLOC_016136	PSEN1	14:73136251-73223797
XLOC_016176	FOS	14:75278511-75397025
XLOC_016178	JDP2	14:75423682-75474168
XLOC_016180	FLVCR2,IFT43,TTLL5	14:75574887-76084585
XLOC_016233	CTD-2341M24.1	14:85934613-86160901
XLOC_016290	SLC24A4	14:92322580-92509155
XLOC_016347	CTD-2506J14.1	14:97458815-97581601
XLOC_016348	RP11-204N11.1	14:97706147-97775075
XLOC_016367	EVL	14:99971448-100167219
XLOC_016383	-	14:100683747-100689596
XLOC_016510	RCOR1	14:102592660-102730576
XLOC_016542	TDRD9	14:103928455-104052667
XLOC_016570	CRIP2	14:105472961-105480321
XLOC_016572	TMEM121	14:105526602-105530202
XLOC_016660	MIR6717,NDRG2	14:21016762-21104722
XLOC_016675	SALL2	14:21521080-21537216
XLOC_016680	OR10G2	14:21632338-21636222
XLOC_016705	SLC7A7	14:22702142-22829820
XLOC_016712	AJUBA,HAUS4,MIR4707,RP11-298I3.5	14:22929608-22982642
XLOC_016715	CDH24	14:23047061-23057581
XLOC_016717	CEBPE	14:23117303-23120265
XLOC_016747	CTSG	14:24573521-24576914
XLOC_016748	GZMH	14:24595722-24657774
XLOC_016749	GZMB	14:24595722-24657774
XLOC_016804	SPTSSA	14:33924230-34462774
XLOC_016813	BAZ1A	14:34752671-34876705
XLOC_016823	NFKBIA	14:35397754-35405287
XLOC_016906	RPL32P29,RPS29	14:49543094-49614824
XLOC_016933	PYGL	14:50848121-50944736
XLOC_016953	ERO1L	14:52639825-52695944
XLOC_016968	CNIH1	14:54423232-54441442
XLOC_016969	GMFB	14:54474399-54489196
XLOC_016978	DLGAP5	14:55148111-55191678
XLOC_017049	WDR89	14:63589702-63652079
XLOC_017104	ZFP36L1	14:68787436-68796458

XLOC_017106	ACTN1,HMGN1P3	14:68870327-68987463
XLOC_017148	NUMB,RP1-240K6.3,RP4-647C14.3	14:73237496-73477175
XLOC_017156	PNMA1	14:73711782-73714372
XLOC_017173	LTBP2,MIR4709,NPC2	14:74476191-74612378
XLOC_017217	TMED8	14:77334760-77391497
XLOC_017221	SPTLC2	14:77505869-77616878
XLOC_017295	GPR68	14:90847860-91259323
XLOC_017296	CCDC88C	14:91271185-91417901
XLOC_017299	CATSPERB,TC2N	14:91580695-91867721
XLOC_017304	FBLN5	14:91869409-91947987
XLOC_017325	DDX24	14:94026328-94103846
XLOC_017327	SERPINA1	14:94130563-94390772
XLOC_017341	CLMN,CTD-2240H23.2	14:95181937-95319906
XLOC_017362	LINC01550	14:97925609-97978281
XLOC_017371	BCL11B	14:99169234-99272310
XLOC_017372	-	14:99279784-99287503
XLOC_017386	WARS	14:100333787-100530303
XLOC_017418	CDC42BPB	14:102922655-103057479
XLOC_017427	CKB	14:103519658-103523111
XLOC_017434	PPP1R13B	14:103733751-103847590
XLOC_017458	-	14:105075347-105089606
XLOC_017600	IGHV3-74	14:106810374-106811131
XLOC_017659	-	14:99052937-99059398
XLOC_017660	-	14:99059570-99063424
XLOC_017661	-	14:99063497-99066486
XLOC_017728	RP11-603B24.1	15:22253527-22297735
XLOC_017736	WHAMMP3	15:22664358-22687892
XLOC_017739	NIPA1,RP11-566K19.6	15:22757856-22829791
XLOC_017751	MKRN3	15:23565677-23630075
XLOC_017760	PWAR5,PWAR6,RP11-701H24.7,SNHG14,SNORD107,SNORD115-13,SNORD115-26,SNORD115-46,SNORD115-7,SNORD116,SNORD116-20,SNORD116-22,SNORD116-3,SNORD116-4,SNORD116-5,SNORD64,SNRPN,SNURF	15:24823646-25439094
XLOC_017863	WHAMMP2	15:28701393-28764438
XLOC_017865	APBA2	15:28884482-29118315
XLOC_017869	HMGN2P5	15:29699366-29968865
XLOC_017939	GOLGA8B	15:34525085-34588503
XLOC_017979	THBS1	15:39580370-39599905
XLOC_018001	BAHD1	15:40439720-40468242
XLOC_018006	CASC5	15:40594019-40664342
XLOC_018013	SPINT1	15:40835807-40858207
XLOC_018025	CHP1	15:41230838-41281927
XLOC_018027	NUSAP1	15:41332693-41381050
XLOC_018093	BLOC1S6,HMGN2P46,RP11-96O20.4,SQRDL	15:45430528-45691294
XLOC_018199	AQP9	15:57953423-58569843
XLOC_018229	RPS3AP6	15:59768351-59769146

XLOC_018261	LACTB	15:63121799-63158021
XLOC_018265	USP3	15:63500736-63602428
XLOC_018282	AC069368.3,ANKDD1A,PLEKHO2	15:64841882-64990310
XLOC_018311	SMAD3	15:67063762-67195195
XLOC_018333	KIF23	15:69298946-69448427
XLOC_018394	SCAMP5	15:74954415-75021496
XLOC_018399	C15orf39	15:75195642-75213743
XLOC_018472	ST20-AS1	15:79833584-79927152
XLOC_018474	BCL2A1	15:79960888-80017858
XLOC_018488	IL16	15:81159574-81324183
XLOC_018571	ABHD2	15:89087077-89202386
XLOC_018576	LINC00925	15:89361578-89398487
XLOC_018586	ANPEP	15:89784888-89815419
XLOC_018600	IQGAP1	15:90249529-90502253
XLOC_018610	FURIN	15:90868591-90883629
XLOC_018611	FES,MAN2A2	15:90883694-90922584
XLOC_018620	SLCO3A1	15:91852977-92331037
XLOC_018672	ARRDC4	15:97960697-98021777
XLOC_018795	CYFIP1	15:22838640-22981063
XLOC_018820	ATP10A	15:25673178-25865172
XLOC_018917	LPCAT4	15:34358617-34367704
XLOC_018919	GOLGA8A	15:34379067-34437498
XLOC_018950	RASGRP1	15:38453966-38565575
XLOC_018957	CTD-2033D15.1,CTD-2033D15.3	15:39580370-39599905
XLOC_018964	BMF	15:40087889-40108899
XLOC_018998	LTK	15:41503637-41513900
XLOC_019097	FBN1	15:48408305-48645849
XLOC_019112	ATP8B4	15:49857986-50236395
XLOC_019126	DMXL2	15:51414096-51625289
XLOC_019141	GNB5	15:52115104-52295798
XLOC_019158	PIGBOS1,RAB27A	15:55202965-55508234
XLOC_019160	CCPG1,DYX1C1,DYX1C1-CCPG1,<br>MIR628	15:55202965-55508234
XLOC_019166	NEDD4	15:55826921-55993746
XLOC_019188	ALDH1A2	15:57953423-58569843
XLOC_019191	AQP9	15:57953423-58569843
XLOC_019216	BNIP2	15:59594874-59690575
XLOC_019217	-	15:59690672-59696227
XLOC_019221	ANXA2	15:60347133-60402883
XLOC_019224	RORA,uc_338	15:60479177-61229319
XLOC_019249	-	15:63488370-63498038
XLOC_019254	DAPK2	15:63907035-64072033
XLOC_019255	FAM96A	15:64072558-64094018
XLOC_019262	OAZ2	15:64687572-64719602
XLOC_019333	UACA	15:70654553-70763593
XLOC_019350	PKM	15:72199028-72233377
XLOC_019360	ADPGK	15:72751364-72798199
XLOC_019367	NPTN	15:73443157-73634134
XLOC_019414	IMP3	15:75636138-75662336
XLOC_019463	CTSH	15:78900341-78949574

XLOC_019464	RASGRF1	15:78959946-79090773
XLOC_019474	BCL2A1	15:79960888-80017858
XLOC_019501	AC245033.1,GOLGA2P10	15:82459471-82513965
XLOC_019533	SEC11A	15:84669537-84721164
XLOC_019570	HAPLN3	15:88877287-88895629
XLOC_019571	MFGE8	15:88898682-88913435
XLOC_019589	ANPEP	15:89784888-89815419
XLOC_019701	CHSY1	15:101175499-101252637
XLOC_019711	TARSL2	15:101653597-101725987
XLOC_019747	-	15:66843035-66846404
XLOC_019780	HBA2	16:172846-173710
XLOC_019815	CACNA1H	16:1153240-1225257
XLOC_019830	TMEM204	16:1485885-1612110
XLOC_019893	LA16c-380H5.5	16:3006119-3014505
XLOC_019898	MMP25	16:3022619-3061081
XLOC_019899	IL32,RNU1-125P,RNU1-22P	16:3064566-3087704
XLOC_019931	HMOX2	16:4461659-4510612
XLOC_019967	C16orf72,RP11-473I1.9	16:9091604-9121640
XLOC_020079	CTD-2349B8.1,ITPRIPL2,SYT17	16:19113388-19268334
XLOC_020111	METTL9	16:21597217-21657598
XLOC_020148	PRKCB	16:23835943-24220611
XLOC_020178	IL4R	16:27313386-27364895
XLOC_020179	IL21R	16:27402161-27453393
XLOC_020188	SBK1	16:28241267-28331934
XLOC_020194	APOBR	16:28456371-28512051
XLOC_020208	LAT,RP11-264B17.3,SPNS1	16:28973845-28990783
XLOC_020221	AC009133.22,QPRT,SPN	16:29662789-29735401
XLOC_020233	ALDOA	16:30053089-30070457
XLOC_020252	ITGAL	16:30472653-30523523
XLOC_020284	ITGAM	16:31259917-31336040
XLOC_020285	ITGAX	16:31354678-31384230
XLOC_020501	RBL2	16:53433976-53504411
XLOC_020515	LPCAT2,RP11-212I21.4	16:55508997-55586670
XLOC_020550	CPNE2	16:57092403-57152166
XLOC_020561	GPR114,HMGB3P32	16:57542420-57607359
XLOC_020562	GPR56	16:57610651-57665741
XLOC_020563	GPR97	16:57668186-57731805
XLOC_020578	NDRG4	16:58462845-58513756
XLOC_020613	LINC00920	16:66408509-66413158
XLOC_020624	PDP2,RP11-61A14.2,RP11-61A14.3	16:66878588-66895754
XLOC_020631	NOL3	16:67170153-67190204
XLOC_020642	RLTPR	16:67644889-67660815
XLOC_020656	PLA2G15	16:68245303-68261406
XLOC_020729	HP,HPR	16:72008522-72176878
XLOC_020849	ATP2C2	16:84368494-84467361
XLOC_020855	CRISPLD2	16:84819983-84939603
XLOC_020860	KIAA0513	16:85027750-85094393
XLOC_020914	ZFPM1	16:88453316-88537287
XLOC_020916	-	16:88540059-88560351
XLOC_020926	CDT1	16:88803212-88809258

XLOC_020950	VPS9D1-AS1,ZNF276	16:89707133-89938761
XLOC_020956	AC137934.1,FAM157C	16:90101003-90222678
XLOC_020964	AXIN1	16:287439-355394
XLOC_020968	RAB11FIP3	16:425618-523079
XLOC_021025	MSRB1	16:1935541-1943466
XLOC_021044	ABCA3	16:2275880-2426699
XLOC_021067	PRSS30P	16:2830168-2843189
XLOC_021072	PKMYT1	16:2963943-2980539
XLOC_021079	RP11-473M20.7	16:3022619-3061081
XLOC_021086	MEFV	16:3162131-3256955
XLOC_021098	NLRC3	16:3365098-3577774
XLOC_021100	RP11-461A8.4,TRAP1	16:3611662-3717597
XLOC_021112	CDIP1	16:4510674-4538861
XLOC_021213	CPPED1	16:12651736-12804017
XLOC_021324	IGSF6	16:21597217-21657598
XLOC_021387	SBK1	16:28241267-28331934
XLOC_021455	RP11-297C4.6,SEPT1	16:30378094-30400121
XLOC_021493	PYCARD	16:31199236-31227160
XLOC_021607	HMGN2P41	16:35802146-35803280
XLOC_021683	RP11-44F14.1,RP11-44F14.4	16:53362018-53373083
XLOC_021706	CES1	16:55802850-55833337
XLOC_021732	CCDC102A	16:57512177-57537132
XLOC_021733	GPR114	16:57542420-57607359
XLOC_021735	GPR56	16:57610651-57665741
XLOC_021801	NAE1	16:66802874-66873256
XLOC_021818	ATP6V0D1	16:67438013-67546973
XLOC_021935	MLKL	16:74671854-74701181
XLOC_021988	CDYL2	16:80597905-80804380
XLOC_022031	COTL1	16:84565488-84618077
XLOC_022074	KLHDC4,RP11-278A23.1	16:87600525-87766163
XLOC_022255	P2RX1	17:3860314-3918577
XLOC_022264	SPNS2	17:4497393-4555631
XLOC_022271	ARRB2	17:4710365-4721720
XLOC_022316	ASGR2	17:7074479-7115700
XLOC_022317	RPL7AP64	17:7131257-7179564
XLOC_022329	NLGN2	17:7404840-7419860
XLOC_022334	CD68,EIF4A1,MPDU1,SENP3,SENP3-EIF4A1,SNORA48,SNORA67,SNORD10,TNFSF12,TNFSF12-TNFSF13,TNFSF13	17:7548483-7633383
XLOC_022341	KDM6B,TMEM88	17:7834202-7856099
XLOC_022343	CHD3	17:7856683-7912960
XLOC_022408	HS3ST3B1	17:14301082-14349404
XLOC_022450	CCDC144A,NOS2P4,RP11-219A15.1,RP11-219A15.2,RP11-219A15.4,USP32P1	17:16689536-16816540
XLOC_022494	KRT16P4,LGALS9C	17:18450243-18494945
XLOC_022549	CCDC144CP,SPECC1,USP32P3	17:20009343-20433062
XLOC_022616	RP11-173M1.7,RP11-173M1.8,WSB1	17:27294075-27348491

XLOC_022684	CPD	17:30378904-30469989
XLOC_022699	ADAP2,RN7SL138P,RNF135,RP11-848P1.3	17:30831969-31000180
XLOC_022726	RHOT1	17:32141225-32253374
XLOC_022769	SLFN5	17:35223597-35273656
XLOC_022792	AC015849.16,RDM1	17:35893706-35965472
XLOC_022827	DUSP14	17:37489830-37513501
XLOC_022839	CISD3,CTB-58E17.1,MLLT6	17:38701680-38751457
XLOC_022865	ERBB2	17:39671121-39730787
XLOC_022868	IKZF3	17:39757519-39864624
XLOC_022876	THRA	17:40019096-40100767
XLOC_022884	RARA	17:40309191-40357833
XLOC_022899	-	17:41108245-41115819
XLOC_022929	ATP6V0A1,MIR5010,RP11-400F19.18	17:42458843-42522611
XLOC_022987	GRN	17:44345115-44353128
XLOC_023048	TBX21	17:47733192-47746652
XLOC_023116	ABCC3	17:50634626-50692252
XLOC_023150	PCTP	17:55750978-55872939
XLOC_023157	NOG	17:56593698-56595590
XLOC_023164	SCPEP1	17:56978104-57006768
XLOC_023178	DYNLL2,RP11-159D12.8	17:58083392-58191948
XLOC_023200	YPEL2	17:59331617-59403303
XLOC_023204	CLTC,VMP1	17:59618552-59844367
XLOC_023214	CA4	17:60149935-60170899
XLOC_023270	MILR1	17:64449019-64474114
XLOC_023285	RGS9	17:65137374-65227703
XLOC_023329	ARSG,PRKAR1A,RP11-120M18.2	17:68259181-68763882
XLOC_023343	KCNJ2	17:70163972-70180048
XLOC_023364	RPL38	17:74203317-74213342
XLOC_023367	KIF19	17:74326209-74355820
XLOC_023375	MIR3615,RAB37,SLC9A3R1	17:74596985-74769360
XLOC_023397	LLGL2,TSEN54	17:75516059-75575208
XLOC_023435	SCARNA16,SEC14L1,SNHG20	17:77086715-77217101
XLOC_023436	SEPT9	17:77257736-77500697
XLOC_023448	TMC8	17:77959256-78143110
XLOC_023451	SYNGR2	17:78168557-78207701
XLOC_023453	BIRC5	17:78214185-78225636
XLOC_023478	GAA	17:80101338-80119909
XLOC_023489	RPTOR	17:80544818-80971213
XLOC_023530	MIR6787,RP13-516M14.10,RP13-516M14.8,SLC16A3	17:82228396-82276189
XLOC_023556	RP11-1228E12.1	17:64098-76866
XLOC_023559	DOC2B	17:142788-181636
XLOC_023564	FAM101B	17:439977-492206
XLOC_023575	YWHAE	17:1344271-1400378
XLOC_023576	CRK	17:1420688-1463162
XLOC_023580	SLC43A2	17:1567132-1632757
XLOC_023582	RILP,SCARF1	17:1633651-1650077
XLOC_023585	MIR22,MIR22HG	17:1711492-1755273

XLOC_023628	RP11-147K16.3,SPATA22,TRPV3	17:3439922-3557995
XLOC_023635	CAMKK1,P2RX1	17:3860314-3918577
XLOC_023648	PELP1	17:4669256-4705529
XLOC_023651	CXCL16,RP11-314A20.5	17:4731406-4746119
XLOC_023669	SCIMP	17:5192083-5248069
XLOC_023692	ASGR2,CLEC10A	17:7074479-7115700
XLOC_023707	ZBTB4	17:7459329-7484263
XLOC_023733	AURKB	17:8204732-8212228
XLOC_023757	GAS7	17:9910455-10198898
XLOC_023798	PMP22	17:15229776-15265707
XLOC_023833	TBC1D27,TNFRSF13B	17:16922776-16972242
XLOC_023863	CCDC144B,CTD-2303H24.2,FAM106A,USP32P2	17:18510836-18625647
XLOC_023865	FOXO3B,RP11-815I9.3,RP11-815I9.4,TBC1D28,ZNF286B	17:18635005-18682411
XLOC_023994	RAB34	17:28714262-28718429
XLOC_024061	CTD-2370N5.3,EVI2A,EVI2B	17:31008153-31382116
XLOC_024119	RP11-1094M14.5,RP11-686D22.10,SLFN12L,SLFN13,TOMM20P2	17:35435095-35538497
XLOC_024130	HEATR9	17:35568119-35885916
XLOC_024132	CCL5	17:35568119-35885916
XLOC_024159	SYNRG	17:37514796-37609496
XLOC_024193	PLXDC1	17:39057018-39154394
XLOC_024207	IKZF3,RP11-94L15.2	17:39757519-39864624
XLOC_024210	GSDMB,ORMDL3	17:39904405-39939601
XLOC_024222	TOP2A	17:40388420-40417950
XLOC_024228	CCR7	17:40548316-40565509
XLOC_024241	KRT23	17:40921429-40975926
XLOC_024291	JUP	17:41754603-41786931
XLOC_024292	LEPREL4	17:41801946-41823217
XLOC_024310	STAT3	17:42313323-42388606
XLOC_024327	LINC00671	17:42865921-42898704
XLOC_024332	VAT1	17:43006645-43025123
XLOC_024333	BRCA1	17:43035594-43125483
XLOC_024359	DUSP3	17:43765513-43779025
XLOC_024426	WNT3	17:46762505-46833154
XLOC_024444	TBX21	17:47733192-47746652
XLOC_024445	OSBPL7	17:47807371-47821834
XLOC_024463	SKAP1	17:48103356-48430307
XLOC_024525	LRRC59	17:50373219-50397888
XLOC_024567	MIR3614,TRIM25	17:56791912-56914738
XLOC_024579	CUEDC1	17:57861242-57955352
XLOC_024586	MPO	17:58192736-58280935
XLOC_024674	CYB561,RP11-269G24.4	17:62935401-63447173
XLOC_024687	ICAM2	17:63996070-64020634
XLOC_024689	TEX2	17:64147226-64263301
XLOC_024691	PECAM1	17:64319414-64413896
XLOC_024710	AXIN2,CEP112,CTD-2535L24.2	17:65528562-66192084
XLOC_024736	FAM20A	17:68259181-68763882
XLOC_024740	ABCA6,ABCA9	17:68974487-69141888

XLOC_024767	CDC42EP4	17:73282830-73312175
XLOC_024780	CD300LB	17:74519052-74546938
XLOC_024781	CD300C	17:74519052-74546938
XLOC_024803	GRB2	17:75318075-75405709
XLOC_024816	TRIM65	17:75879867-75897021
XLOC_024818	ACOX1	17:75941506-76027452
XLOC_024860	SOCS3	17:78356777-78360077
XLOC_024864	CTD-2357A8.3	17:78617388-78632322
XLOC_024865	CYTH1	17:78671576-78782356
XLOC_024868	USP36	17:78787312-78841441
XLOC_024870	KIAA1731NL,TIMP2	17:78852485-78925387
XLOC_024900	BAIAP2-AS1	17:81029065-81034881
XLOC_024901	AATK	17:81035121-81183164
XLOC_024908	LINC00482	17:81302754-81309408
XLOC_024913	RP11-1055B8.4	17:81388125-81466332
XLOC_024945	-	17:82217868-82220413
XLOC_024962	RAB40B	17:82654972-82698728
XLOC_025037	-	17:66301078-66302492
XLOC_025055	-	17:77863895-77867395
XLOC_025059	-	17:82221307-82222700
XLOC_025092	EMILIN2,RP11-737O24.5	18:2846474-3013367
XLOC_025162	RAB31	18:9708164-9862716
XLOC_025165	VAPA	18:9912315-10020287
XLOC_025190	IMPA2	18:11981024-12033467
XLOC_025227	ANKRD20A5P,CYP4F35P,RHOT1P1,RP11-757O6.1,RP11-757O6.6	18:14179096-14342525
XLOC_025269	RIOK3	18:23452822-23486603
XLOC_025350	MAPRE2	18:34976927-35143470
XLOC_025393	SIGLEC15	18:45825511-45844818
XLOC_025419	CTIF,MIR4743	18:48539045-48863217
XLOC_025456	RAB27B	18:54717859-54898083
XLOC_025507	PMAIP1	18:59899947-59904306
XLOC_025544	SERPINB10,SERPINB2	18:63871691-63936111
XLOC_025545	HMSD,SERPINB8	18:63949300-64005667
XLOC_025604	TSHZ1	18:75210623-75289991
XLOC_025646	KCNG2	18:79792725-79900400
XLOC_025667	ENOSF1	18:596987-712676
XLOC_025668	YES1	18:721587-813756
XLOC_025711	ZBTB14	18:5289018-5297053
XLOC_025713	EPB41L3	18:5392380-5630700
XLOC_025788	RP11-973H7.1,RP11-973H7.4	18:12738964-12776351
XLOC_025866	RP11-535A5.1	18:22345239-22349482
XLOC_025930	DSC2	18:31058839-31162979
XLOC_026002	PSTPIP2	18:45983436-46072292
XLOC_026007	LOXHD1	18:46326808-46657033
XLOC_026034	SMAD7	18:48919852-48952043
XLOC_026057	MEX3C	18:51174549-51218304
XLOC_026135	BCL2	18:63123212-63320273
XLOC_026167	CD226,DOK6,RP11-543H23.2	18:69401054-69961803
XLOC_026204	ZNF516	18:76357598-76498088

XLOC_026276	GZMM	19:544033-549924
XLOC_026277	-	19:550278-551744
XLOC_026284	FSTL3	19:676364-683408
XLOC_026291	AZU1	19:825096-832050
XLOC_026292	PRTN3	19:840959-848175
XLOC_026293	ELANE	19:851013-856247
XLOC_026298	ARID3A	19:925744-976093
XLOC_026312	MIDN	19:1248501-1259305
XLOC_026346	GADD45B	19:2476121-2478259
XLOC_026360	GNA15	19:3136192-3163802
XLOC_026373	AC005954.4,TJP3	19:3708108-3761703
XLOC_026375	MATK	19:3777855-3802129
XLOC_026390	FSD1	19:4304159-4323843
XLOC_026401	SEMA6B	19:4542057-4559808
XLOC_026438	TNFSF9	19:6530998-6535928
XLOC_026443	EMR1	19:6887529-6942813
XLOC_026456	RETN	19:7655875-7670463
XLOC_026458	CTD-3214H19.16,MCEMP1,TRAPPC5	19:7676627-7688051
XLOC_026501	CTC-325H20.2,ZNF177,ZNF559,ZNF559-ZNF177	19:9323771-9383221
XLOC_026530	ICAM1	19:10251900-10289019
XLOC_026541	QTRT1	19:10701420-10713437
XLOC_026546	C19orf38	19:10717900-10869790
XLOC_026579	ZNF788	19:12092262-12156751
XLOC_026602	JUNB	19:12763002-12874951
XLOC_026668	CYP4F22	19:15508414-15552325
XLOC_026671	CYP4F3,CYP4F8	19:15615217-15662825
XLOC_026723	COLGALT1	19:17554061-17586372
XLOC_026731	RPL18A,SNORA68	19:17859875-17864153
XLOC_026742	IFI30,PIK3R2	19:18152872-18178118
XLOC_026746	LSM4	19:18303448-18323274
XLOC_026769	SLC25A42	19:19063998-19112953
XLOC_026899	PLEKHF1	19:29665055-29675478
XLOC_026904	URI1	19:29923643-30016750
XLOC_026924	ZNF507	19:32345593-32387800
XLOC_026925	DPY19L3	19:32390049-32485895
XLOC_026961	ZNF181	19:34734084-34745378
XLOC_026966	ZNF30	19:34923298-34945303
XLOC_026984	FFAR2	19:35443906-35452360
XLOC_026988	HAUS5	19:35612743-35625907
XLOC_027001	LRFN3	19:35935357-35945767
XLOC_027028	ZNF420	19:37007856-37249046
XLOC_027032	HKR1	19:37312836-37369365
XLOC_027082	ZFP36	19:39400916-39409719
XLOC_027115	LTBP4	19:40592882-40629818
XLOC_027136	AC005795.1	19:41506151-41506898
XLOC_027141	CEACAM3	19:41796427-41812121
XLOC_027148	ZNF574	19:41998320-42081565
XLOC_027163	CD177	19:43353508-43368970

XLOC_027196	BCL3,MIR8085	19:44747704-44760188
XLOC_027218	PPM1N	19:45485288-45503364
XLOC_027219	VASP	19:45506240-45602212
XLOC_027222	GIPR	19:45668189-45683882
XLOC_027259	C5AR1	19:47290022-47322069
XLOC_027260	C5AR2,DHX34	19:47332146-47382715
XLOC_027292	PPP1R15A	19:48872391-48876278
XLOC_027296	CTD-2639E6.9,FTL	19:48963444-48967908
XLOC_027317	ALDH16A1,CTD-3148I10.15,CTD-3148I10.9,FLT3LG,RPL13A,SNORD32A,SNORD33,SNORD34,SNORD35A	19:49446202-49492308
XLOC_027319	FCGRT,RCN3	19:49506815-49546962
XLOC_027324	ADM5,MIR5088,PRMT1	19:49675785-49690575
XLOC_027347	CLEC11A	19:50723328-50725718
XLOC_027360	CTD-3187F8.15,SIGLEC18P,SIGLEC7,SIGLEC9	19:51119986-51181966
XLOC_027372	CTD-2616J11.14,CTD-2616J11.2,CTD-2616J11.3,LIM2	19:51379908-51420324
XLOC_027386	FPR1,FPR2	19:51740744-51826189
XLOC_027503	LILRA5	19:54300425-54339356
XLOC_027516	AC009892.9,AC011515.2,CTB-83J4.2,LILRA1,LILRA2,LILRB1,LILRB4,LILRP1,VN1R105P	19:54572917-54700954
XLOC_027526	CTB-61M7.1,KIR2DL1,KIR2DL3,KIR2DL4,KIR2DP1,KIR2DS4,KIR3DL1,KIR3DL2,KIR3DL3,KIR3DP1	19:54724456-54867542
XLOC_027527	CTB-61M7.2,FCAR	19:54874163-54898135
XLOC_027528	NCR1	19:54905896-54920010
XLOC_027592	AC003002.4,AC003002.6,AC003005.4,AC004076.7,TRAPPC2B,ZNF17,ZNF419,ZNF547,ZNF548,ZNF749,ZNF773	19:57363443-57518480
XLOC_027629	RPS5	19:58384654-58394823
XLOC_027638	TRIM28	19:58544069-58550722
XLOC_027647	AC098474.1,LINC01002	19:197954-246515
XLOC_027679	SBNO2	19:1107635-1174291
XLOC_027688	PCSK4	19:1481427-1497927
XLOC_027699	ABHD17A	19:1876809-1885682
XLOC_027735	TLE2	19:2997637-3047635
XLOC_027736	AES	19:3052909-3063118
XLOC_027740	-	19:3164111-3170582
XLOC_027745	NFIC	19:3359562-3469247
XLOC_027759	MATK	19:3777855-3802129
XLOC_027771	TMIGD2	19:4292231-4302431
XLOC_027781	CTB-50L17.14,LRG1,PLIN5	19:4518273-4541821
XLOC_027782	SEMA6B	19:4542057-4559808
XLOC_027790	PLIN3,UHRF1	19:4838307-5153616
XLOC_027794	PTPRS	19:5158494-5340803
XLOC_027817	DENND1C	19:6436078-6482557

XLOC_027818	TUBB4A	19:6494318-6502848
XLOC_027827	CTD-3128G10.6,SH2D3A	19:6737924-6767659
XLOC_027835	INSR	19:7112254-7294341
XLOC_027865	PRAM1	19:8490015-8504674
XLOC_027867	MYO1F	19:8517341-8577577
XLOC_027869	ADAMTS10	19:8580025-8610745
XLOC_027904	OLFM2	19:9853717-9936552
XLOC_027917	S1PR5	19:10512485-10517931
XLOC_027919	KRI1	19:10543720-10566110
XLOC_027943	ZNF823	19:11721212-11739009
XLOC_027992	CTD-3252C9.4,MIR23A,MIR24-2,MIR27A	19:13780069-13842906
XLOC_028001	PRKACA	19:14087839-14118084
XLOC_028009	GIPC1	19:14477759-14496324
XLOC_028012	EMR3	19:14583083-14778541
XLOC_028014	EMR2	19:14583083-14778541
XLOC_028027	NOTCH3	19:15159037-15200981
XLOC_028037	RASAL3	19:15450101-15465808
XLOC_028109	LRRC25	19:18389610-18398527
XLOC_028110	ISYNA1	19:18418543-18522127
XLOC_028135	PBX4	19:19561668-19618916
XLOC_028160	CTC-513N18.7,CTD-2542C24.3,ZNF626,ZNF737,ZNF826P	19:20340268-20863281
XLOC_028222	ZNF91	19:23304990-23395560
XLOC_028261	C19orf12	19:29691981-29715789
XLOC_028268	TSHZ3	19:31242777-31417794
XLOC_028290	CEP89,CTD-2085J24.4	19:32875870-32972020
XLOC_028295	CEBPA	19:33299804-33302581
XLOC_028313	CTC-523E23.1,CTC-523E23.11,CTC-523E23.4,ZNF599	19:34757478-34860684
XLOC_028320	ZNF792	19:34955518-34964396
XLOC_028339	DMKN	19:35497219-35513658
XLOC_028393	CTD-2528L19.4,ZFP30,ZNF607,ZNF781	19:37497158-37719790
XLOC_028406	RASGRP4	19:38403104-38426305
XLOC_028431	RPS16	19:39433142-39435948
XLOC_028437	CLC	19:39731249-39738369
XLOC_028439	DYRK1B,FBL	19:39825349-39846415
XLOC_028441	FCGBP	19:39854456-39953459
XLOC_028459	BLVRB	19:40447788-40465840
XLOC_028484	AC011513.4,CEACAM4	19:41545191-41786893
XLOC_028492	ATP1A3	19:41966581-41997497
XLOC_028503	CEACAM1	19:42397127-43207299
XLOC_028505	CEACAM8,PSG3	19:42397127-43207299
XLOC_028527	PLAUR	19:43645641-43695034
XLOC_028553	BCL3	19:44747704-44760188
XLOC_028577	AC011530.4,DMPK,DMWD	19:45764784-45792864
XLOC_028580	IRF2BP1	19:45883606-45886170
XLOC_028584	PGLYRP1	19:45995443-46030161
XLOC_028692	NOSIP	19:49555178-49628266

XLOC_028747	CLDND2,CTD-2616J11.11,ETFB,NKG7	19:51345152-51373869
XLOC_028754	SIGLEC12	19:51491216-51501789
XLOC_028763	FPR1	19:51740744-51826189
XLOC_028815	NLRP12	19:53630887-53991601
XLOC_028818	VSTM1	19:54040824-54063999
XLOC_028819	TARM1	19:54069381-54081445
XLOC_028820	OSCAR	19:54094626-54131719
XLOC_028824	MBOAT7	19:54173411-54194536
XLOC_028827	LILRA6,LILRB2,LILRB3	19:54200741-54281212
XLOC_028834	AC008984.2,AC008984.6,LILRA4,LILRA5,VN1R104P	19:54300425-54339356
XLOC_028847	NCR1	19:54905896-54920010
XLOC_028854	TNNT1	19:55132777-55149354
XLOC_028921	ZNF550	19:57527324-57559941
XLOC_028936	ZNF329	19:58126251-58155110
XLOC_028992	-	19:44746004-44747470
XLOC_029042	RNF144A	2:6912276-7077880
XLOC_029071	C2orf48,RRM2	2:10122327-10212792
XLOC_029096	LPIN1	2:11677594-11827560
XLOC_029103	TRIB2	2:12716888-12742739
XLOC_029146	RHOB	2:20447073-20449445
XLOC_029188	RAB10	2:26033822-26138968
XLOC_029199	CENPA,SLC35F6	2:26764253-26801067
XLOC_029232	PLB1	2:28457144-28664540
XLOC_029239	FAM179A	2:28956610-29074261
XLOC_029244	YPEL5	2:30146940-30160557
XLOC_029247	LBH	2:30231530-30323730
XLOC_029296	VIT	2:36696689-36814792
XLOC_029303	QPCT	2:37344573-37373322
XLOC_029307	CYP1B1-AS1,RMDN2	2:37923170-38181855
XLOC_029313	RPLP0P6	2:38406718-38515740
XLOC_029323	SLC8A1-AS1	2:39786452-40611053
XLOC_029371	RHOQ	2:46490749-46684746
XLOC_029384	MSH2	2:47402968-47570939
XLOC_029422	RPS27A	2:55172600-55271145
XLOC_029438	EIF3FP3	2:58251439-58252525
XLOC_029491	LGALSL	2:64453968-64461381
XLOC_029506	ACTR2	2:65227752-65271253
XLOC_029529	ARHGAP25	2:68679600-68826852
XLOC_029541	MXD1,SNRNP27	2:69893559-69942963
XLOC_029567	NAGK	2:70935881-71081674
XLOC_029572	DYSF	2:71452783-71686815
XLOC_029594	AC073046.25,TET3	2:73984787-74108176
XLOC_029603	WDR54	2:74421677-74442422
XLOC_029610	SEMA4F	2:74654227-74693172
XLOC_029613	HK2	2:74832654-74893463
XLOC_029678	GNLY	2:85685143-85700539
XLOC_029687	KDM3A	2:86440646-86492821
XLOC_029788	MAL	2:95023448-95077047

XLOC_029815	ITPRIPL1	2:96324887-96330805
XLOC_029830	ANKRD36	2:97113115-97265736
XLOC_029845	ZAP70	2:97712321-97996374
XLOC_029880	-	2:101962065-101988700
XLOC_029881	IL1R2	2:101991811-102028544
XLOC_029882	IL1R1	2:102064543-102182108
XLOC_029886	AC007278.3,IL18RAP	2:102418412-102452569
XLOC_029926	CD8BP	2:106487092-106507437
XLOC_029947	LIMS1	2:108534354-108687353
XLOC_029954	SOWAHC	2:109614333-109618990
XLOC_029985	MERTK	2:111898445-112029561
XLOC_029994	POLR1B	2:112541900-112581467
XLOC_030009	IL1RN	2:113107213-113134197
XLOC_030046	STEAP3	2:119223681-119266165
XLOC_030053	PTPN4	2:119759630-119986876
XLOC_030062	RALB	2:120240063-120294716
XLOC_030136	PTPN18	2:130352178-130378557
XLOC_030202	HNMT	2:137964019-138016769
XLOC_030207	AC092620.2,SPOPL	2:138501800-138574458
XLOC_030223	KYNU	2:142877497-143055992
XLOC_030249	KIF5C	2:148866469-149026759
XLOC_030261	TNFAIP6	2:151357591-151380048
XLOC_030286	GPD2	2:156435289-156613735
XLOC_030312	TANK	2:161096230-161254668
XLOC_030317	AC009487.5,SLC4A10,TBR1	2:161416093-161985282
XLOC_030328	GCA	2:162265582-162838730
XLOC_030351	CERS6	2:168455861-168913371
XLOC_030378	CYBRD1	2:171522246-171558133
XLOC_030387	ITGA6	2:172427353-172624111
XLOC_030393	PK	2:173075434-173282036
XLOC_030472	SSFA2	2:181887850-181930822
XLOC_030485	ZNF804A	2:184591879-184939492
XLOC_030611	NOP58,SNORD70	2:202265715-202303670
XLOC_030633	CD28	2:203706474-203778703
XLOC_030636	ICOS	2:203936634-203967940
XLOC_030678	PTH2R	2:208359713-208854503
XLOC_030683	MAP2	2:209424057-209734118
XLOC_030703	SPAG16	2:213284378-214684246
XLOC_030731	IGFBP2	2:216632827-216664436
XLOC_030755	CTDSP1,SLC11A1	2:218381687-218406424
XLOC_030764	CYP27A1,RP11-459I19.1	2:218781492-218832086
XLOC_030776	DNAJB2	2:219279266-219286900
XLOC_030788	SLC4A3	2:219627326-219641980
XLOC_030831	AGFG1,MIR5703	2:227472130-227561417
XLOC_030854	ITM2C	2:230864638-230879248
XLOC_030896	ATG16L1	2:233210050-233295674
XLOC_030919	ARL4C	2:234476111-234497153
XLOC_030923	AGAP1	2:235494088-236131800
XLOC_030941	LRRFIP1,RBM44	2:237627575-237842808
XLOC_030978	ANO7	2:241188508-241225377

XLOC_030983	BOK	2:241544402-241581082
XLOC_031065	LINC00298,LINC00299	2:7922424-8488338
XLOC_031071	MBOAT2	2:8852628-9003880
XLOC_031128	FAM49A	2:16549418-16666331
XLOC_031142	WDR35	2:19910259-20012694
XLOC_031144	LAPTM4A	2:20032649-20052028
XLOC_031258	FAM179A	2:28956610-29074261
XLOC_031269	GALNT14	2:30910466-31155202
XLOC_031282	NLRC4	2:32165840-32265864
XLOC_031319	CDC42EP3	2:37599898-37738468
XLOC_031321	CYP1B1,RMDN2-AS1	2:37923170-38181855
XLOC_031331	DHX57	2:38797728-38875934
XLOC_031345	SLC8A1	2:39786452-40611053
XLOC_031399	C2orf61,CALM2,RP11-761B3.1	2:46899274-47176601
XLOC_031445	RTN4	2:54723498-55112621
XLOC_031452	CCDC88A	2:55287683-55420172
XLOC_031526	PELI1	2:64092651-64257611
XLOC_031546	AC012370.2,SPRED2	2:65310850-65432637
XLOC_031588	MXD1	2:69893559-69942963
XLOC_031598	TGFA	2:70447254-70554193
XLOC_031603	CLEC4F	2:70808531-70820660
XLOC_031612	PAIP2B	2:71182384-71227095
XLOC_031634	MOB1A	2:74152527-74178898
XLOC_031640	RTKN	2:74421677-74442422
XLOC_031646	M1AP	2:74557882-74648338
XLOC_031715	CAPG	2:85394747-85418432
XLOC_031740	CD8A,CD8B	2:86784568-87076719
XLOC_031776	IGKV3-20	2:89142569-89143160
XLOC_031785	IGKV2-29	2:89229768-89235009
XLOC_031862	SEMA4C	2:96859533-96873879
XLOC_031895	CHST10	2:100391859-100417656
XLOC_031958	EDAR	2:108894470-108989372
XLOC_031965	SEPT10	2:109542981-109614270
XLOC_031995	MERTK	2:111898445-112029561
XLOC_031996	MERTK	2:111898445-112029561
XLOC_032006	IL1B	2:112829750-112836903
XLOC_032077	BIN1	2:127048022-127107355
XLOC_032085	LIMS2	2:127638380-127681786
XLOC_032121	CYP4F62P,FAR2P2	2:130416446-130441413
XLOC_032195	CXCR4	2:136114348-136118165
XLOC_032238	RP11-107E5.2,ZEB2	2:144383826-144525011
XLOC_032274	NMI	2:151270464-151290057
XLOC_032308	ACVR1C	2:157526766-157629005
XLOC_032325	CD302,LY75,LY75-CD302	2:159670707-159904749
XLOC_032340	SLC4A10	2:161416093-161985282
XLOC_032341	DPP4	2:161989410-162075169
XLOC_032347	GCA	2:162265582-162838730
XLOC_032353	COBLL1	2:164653623-164849334
XLOC_032371	STK39	2:167954019-168248141
XLOC_032428	RNU6-5P,WIPF1	2:174522826-174774827

XLOC_032475	SESTD1	2:179101691-179264843
XLOC_032503	ZNF804A	2:184591879-184939492
XLOC_032532	SLC40A1	2:189560578-189583758
XLOC_032548	STAT4	2:191021525-191151596
XLOC_032571	STK17B	2:195575976-196176503
XLOC_032672	KLF7	2:207074136-207167338
XLOC_032681	FZD5	2:207762585-207769563
XLOC_032696	IDH1	2:208236226-208266074
XLOC_032708	LANCL1	2:210324758-210477652
XLOC_032714	IKZF2	2:212581356-213154188
XLOC_032789	CHPF	2:219538946-219550595
XLOC_032801	EPHA4	2:221418026-221574454
XLOC_032832	IRS1	2:226731316-226799846
XLOC_032874	NMUR1	2:231501371-231530634
XLOC_032902	HJURP	2:233775678-233854566
XLOC_032909	ARL4C	2:234476111-234497153
XLOC_032975	AC104809.4	2:240906329-240993311
XLOC_033171	-	2:130349982-130351710
XLOC_033229	-	2:227564313-227568717
XLOC_033241	-	2:239501173-239505850
XLOC_033242	-	2:239506059-239507581
XLOC_033268	RAD21L1,SNPH	20:1226055-1309328
XLOC_033271	AL109658.1	20:1441585-1497537
XLOC_033277	SIRPA	20:1888127-1940592
XLOC_033287	EBF4	20:2692877-2760108
XLOC_033304	AP5S1,CDC25B,RP5-1009E24.8,RP5-1009E24.9	20:3786771-3829968
XLOC_033308	FTLP3	20:4023916-4024444
XLOC_033354	ANKEF1,RP5-839B4.8	20:9986087-10368776
XLOC_033416	RIN2,RP1-122P22.2,RP5-999L4.2	20:19756389-20002457
XLOC_033428	XRN2	20:21302730-21389827
XLOC_033461	APMAP,CST7	20:24948939-24992979
XLOC_033503	HM13,MCTS2P	20:31514427-31577923
XLOC_033507	ID1	20:31605282-31606515
XLOC_033511	TPX2	20:31739263-31801805
XLOC_033520	HCK	20:32051486-32103886
XLOC_033556	CHMP4B	20:33811303-33854366
XLOC_033573	PROCR	20:35170948-35177403
XLOC_033582	CEP250	20:35455163-35533998
XLOC_033602	C20orf24,TGIF2,TGIF2-C20orf24	20:36306335-36646375
XLOC_033624	BPI	20:38210487-38337505
XLOC_033636	PPP1R16B	20:38805704-38923024
XLOC_033649	MIR6871,PLCG1,RPL23AP81	20:41028817-41317672
XLOC_033674	OSER1-AS1	20:44210927-44226129
XLOC_033693	-	20:45179843-45192932
XLOC_033711	DNTTIP1	20:45791936-45811429
XLOC_033716	CTSA	20:45881036-45912178
XLOC_033719	MMP9	20:46008907-46060150
XLOC_033762	SNAI1	20:49982998-49988886
XLOC_033765	CEBPB,LINC01273	20:50172549-50193517

XLOC_033767	LINC01272	20:50264210-50290458
XLOC_033768	LINC01270	20:50292719-50321342
XLOC_033802	-	20:53845440-53853598
XLOC_033808	RPL12P4	20:55074567-55075222
XLOC_033862	RP3-492J12.2,ZNF831	20:59123400-59259268
XLOC_033882	RPS21	20:62302067-62388520
XLOC_033894	COL9A3	20:62816243-62861763
XLOC_033919	LIME1,RP4-583P15.10,RP4-583P15.14,RP4-583P15.15,SLC2A4RG,ZGPAT	20:63657809-63831244
XLOC_033922	TPD52L2	20:63865227-63891762
XLOC_033923	AL118506.1,AL118506.2,DNAJC5,MIR941-1,MIR941-2,MIR941-3,MIR941-4	20:63895075-63936554
XLOC_033942	RP5-850E9.3,SCRT2,SRXN1	20:646614-676179
XLOC_033949	FKBP1A,MIR6869,RP11-314N13.10,SDCBP2	20:1309908-1393172
XLOC_033950	NSFL1C,SIRPB2	20:1441585-1497537
XLOC_033951	RP4-576H24.2,RP4-576H24.4,SIRPB1,SIRPD,SIRPG	20:1516758-1657826
XLOC_033986	RNF24,RP4-681N20.5	20:3887281-4015744
XLOC_033995	RASSF2	20:4776288-4825071
XLOC_034012	GPCPD1	20:5544328-5611049
XLOC_034043	JAG1	20:10637556-10994924
XLOC_034091	BFSP1	20:17493904-17569220
XLOC_034161	THBD	20:23045632-23049741
XLOC_034163	CD93	20:23077644-23090136
XLOC_034195	ACSS1	20:25006126-25058980
XLOC_034209	ZNF337	20:25612934-25696846
XLOC_034253	HCK	20:32051486-32103886
XLOC_034256	PLAGL2,TSPY26P	20:32186476-32207791
XLOC_034259	NOL4L	20:32443009-32608893
XLOC_034335	SOGA1	20:36777440-36863686
XLOC_034336	SAMHD1	20:36876120-36951843
XLOC_034371	MAFB	20:40685847-40689240
XLOC_034406	ADA	20:44531784-44652233
XLOC_034407	KCNK15-AS1,LINC01260	20:44656294-44810338
XLOC_034416	SLPI	20:45252238-45254564
XLOC_034447	NCOA5	20:46060984-46089989
XLOC_034482	B4GALT5	20:49363876-49713878
XLOC_034508	ATP9A,NFATC2	20:51386956-51768634
XLOC_034568	CTSZ	20:58981207-59007981
XLOC_034600	WI2-87327B8.2	20:62596202-62605680
XLOC_034629	STMN3	20:63639488-63653427
XLOC_034636	TPD52L2	20:63865227-63891762
XLOC_034652	RP5-968J1.1	20:1804015-1817606
XLOC_034656	-	20:1880065-1883245
XLOC_034684	-	20:53853672-53858822
XLOC_034691	-	20:63850490-63854598
XLOC_034804	CYP4F29P	21:13843132-13980437

XLOC_034846	PPIAP22	21:18857778-18858276
XLOC_034904	AP000240.9,BACH1,BACH1-IT1,BACH1-IT2,GAPDHP14,LINC00189	21:29182026-29940033
XLOC_034931	EVA1C	21:32411123-32515533
XLOC_034948	IFNGR2	21:33402895-33480011
XLOC_034958	KCNE1	21:34418714-34512337
XLOC_034986	TTC3	21:37059169-37203112
XLOC_034992	DSCR8,KCNJ15	21:38121450-38307357
XLOC_034995	ETS2	21:38805306-38824968
XLOC_035034	ABCG1	21:42199688-42304387
XLOC_035036	UBASH3A	21:42403649-42448557
XLOC_035039	PDE9A	21:42653635-42775528
XLOC_035049	RRP1B	21:43659542-43696188
XLOC_035065	TRPM2,snoZ6	21:44350069-44443081
XLOC_035098	COL6A1	21:45981736-46005050
XLOC_035102	COL6A2	21:46098096-46132849
XLOC_035111	DIP2A	21:46458881-46581732
XLOC_035179	ANKRD20A11P,CYP4F29P,RHOT1P2	21:13843132-13980437
XLOC_035186	SAMSN1	21:14485227-14658821
XLOC_035190	AF127936.7	21:14818842-14902950
XLOC_035192	NRIP1	21:14961229-15065897
XLOC_035213	C21orf91,C21orf91-OT1	21:17763314-17819402
XLOC_035264	APP	21:25880549-26217381
XLOC_035268	CYYR1	21:26378551-26573284
XLOC_035269	ADAMTS1	21:26835746-26845409
XLOC_035276	AF131217.1,N6AMT1	21:28439345-28885602
XLOC_035365	AP000322.53,KCNE1	21:34418714-34512337
XLOC_035402	ERG	21:38380026-38661780
XLOC_035443	TMPRSS3	21:42371889-42396846
XLOC_035458	U2AF1	21:43092955-43107711
XLOC_035499	PTTG1IP	21:44849573-44875794
XLOC_035515	SLC19A1	21:45405136-45544411
XLOC_035523	LSS	21:46185078-46228824
XLOC_035529	S100B	21:46598961-46605208
XLOC_035572	-	21:46584702-46593824
XLOC_035613	IL17RA	22:17084953-17115733
XLOC_035674	ZNF74	22:20394114-20413657
XLOC_035733	IGLV8-61	22:22098699-22099212
XLOC_035753	IGLV9-49	22:22343186-22343732
XLOC_035758	IGLV7-46	22:22369468-22370499
XLOC_035766	IGLV1-40	22:22409755-22410307
XLOC_035825	BCR	22:23179703-23318078
XLOC_035861	SGSM1	22:24806168-24927578
XLOC_035862	KIAA1671	22:24952672-25197712
XLOC_035874	ADRBK2	22:25564645-25729294
XLOC_035908	KREMEN1,RNU6-810P,ZNRF3,ZNRF3-IT1,chr22-38_28785274-29006793.1	22:28883591-29197572
XLOC_035922	MTMR3	22:29883113-30177075

XLOC_035939	TCN2	22:30576624-30630664
XLOC_035957	IGLCOR22-1,YWHAH	22:31933520-32203477
XLOC_035995	TOM1	22:35298837-35348069
XLOC_035997	HMOX1	22:35380360-35394214
XLOC_036024	NCF4	22:36847371-36878017
XLOC_036025	CSF2RB	22:36913166-36940486
XLOC_036044	H1F0	22:37805092-37807436
XLOC_036066	APOBEC3F,APOBEC3G	22:39040603-39087743
XLOC_036068	RP4-742C19.12	22:39091022-39092855
XLOC_036079	CACNA1I	22:39570752-39689738
XLOC_036082	GRAP2	22:39900959-39980267
XLOC_036105	TEF	22:41367332-41399405
XLOC_036116	SREBF2	22:41699498-41907308
XLOC_036137	TSPO	22:43151513-43163250
XLOC_036155	ARHGAP8,PRR5,PRR5-ARHGAP8	22:44666740-44862788
XLOC_036178	GRAMD4	22:46576011-46680516
XLOC_036206	PIM3	22:49960434-49993894
XLOC_036210	PANX2	22:50170695-50180294
XLOC_036279	CECR6	22:17116298-17132104
XLOC_036281	CECR1	22:17178789-17221989
XLOC_036304	PI4KAP1	22:18527156-18577968
XLOC_036322	CLTCL1	22:19179430-19291716
XLOC_036330	ARVCF	22:19875516-20016808
XLOC_036336	RTN4R	22:20241414-20283246
XLOC_036374	YPEL1	22:21652269-21735834
XLOC_036378	PPM1F	22:21919236-21982853
XLOC_036504	AC000041.8,AP1B1	22:29327508-29423194
XLOC_036519	OSM	22:30262828-30267401
XLOC_036521	GATSL3,RP1-130H16.18,TBC1D10A	22:30284928-30327046
XLOC_036535	SELM	22:31104771-31120069
XLOC_036542	PIK3IP1	22:31212203-31346232
XLOC_036580	TOM1	22:35298837-35348069
XLOC_036615	IL2RB	22:37125837-37199463
XLOC_036617	RP1-151B14.9,SSTR3	22:37204235-37220721
XLOC_036654	DNAL4,SUN2	22:38705722-38794198
XLOC_036656	NPTXR	22:38818451-38843982
XLOC_036664	CBX7,COX5BP7	22:39120166-39152711
XLOC_036665	PDGFB	22:39223358-39248586
XLOC_036666	RPL3,SNORD43,SNORD83B	22:39312881-39320389
XLOC_036696	RANGAP1	22:41245573-41286251
XLOC_036715	NAGA	22:41998724-42070879
XLOC_036722	NFAM1	22:42159906-42432487
XLOC_036726	RNU6-513P,RRP7B	22:42553616-42582525
XLOC_036756	LDOC1L	22:44492571-44498298
XLOC_036758	PRR5	22:44666740-44862788
XLOC_036762	KIAA0930	22:45189962-45240954
XLOC_036802	Metazoa_SRP	22:49845233-49850896
XLOC_036805	CITF22-49E9.3	22:49932336-49935367
XLOC_036806	PIM3	22:49960434-49993894

XLOC_036809	MLC1	22:50059390-50085902
XLOC_036813	PLXNB2	22:50274874-50307783
XLOC_036815	SBF1	22:50343303-50475069
XLOC_036817	ODF3B,SCO2,TYMP	22:50508215-50534821
XLOC_036892	CHL1	3:195757-409417
XLOC_036952	IRAK2	3:10164864-10243745
XLOC_036960	ATG7,VGLL4	3:11272308-11771350
XLOC_036986	SLC6A6	3:14402550-14542081
XLOC_037012	SATB1-AS1	3:17157161-18917692
XLOC_037061	RP11-222K16.2	3:27712197-27714559
XLOC_037126	ITGA9	3:37452114-37861780
XLOC_037128	CTDSPL	3:37861959-37984469
XLOC_037134	MYD88	3:38138078-38143153
XLOC_037139	ACVR2B	3:38451026-38493142
XLOC_037152	SLC25A38	3:39383080-39397351
XLOC_037153	RPSA,SNORA6,SNORA62	3:39406507-39412542
XLOC_037163	RP11-391M1.4,RPL14	3:40457291-40468587
XLOC_037228	CCR2	3:46353716-46407059
XLOC_037230	CCRL2	3:46407142-46412997
XLOC_037256	CAMP	3:48223346-48225509
XLOC_037301	MAPKAPK3,RP11-804H8.6	3:50606031-51384268
XLOC_037307	GRM2	3:51705728-51718613
XLOC_037317	ALAS1	3:52198085-52214327
XLOC_037324	STAB1	3:52494994-52771027
XLOC_037341	PRKCD	3:53156008-53192717
XLOC_037451	-	3:72738899-72744902
XLOC_037582	TMEM45A	3:100492618-100577445
XLOC_037603	NFKBIZ,NXPE3	3:101779194-101861139
XLOC_037614	ALCAM	3:105366701-105576904
XLOC_037630	-	3:108097942-108122934
XLOC_037637	TRAT1	3:108822522-108861678
XLOC_037651	CD96	3:111292718-111665962
XLOC_037654	PHLDB2,PLCXD2	3:111674385-111976517
XLOC_037673	SIDT1	3:113532295-113631467
XLOC_037677	ATP6V1A	3:113746871-113812058
XLOC_037685	TIGIT	3:114276912-115147319
XLOC_037709	ARHGAP31	3:119294149-119421207
XLOC_037713	ADPRH	3:119579211-119590030
XLOC_037745	CD86	3:122055268-122124001
XLOC_037749	CSTA	3:122325243-122341972
XLOC_037753	PARP15	3:122577517-122639047
XLOC_037758	DIRC2,RP11-67L2.2	3:122794794-122892582
XLOC_037804	CHCHD6	3:126704219-126960488
XLOC_037806	PLXNA1	3:126983087-127037392
XLOC_037821	RAB7A	3:128726121-128814798
XLOC_037831	-	3:129184082-129202860
XLOC_037834	HMCES	3:129278827-129306186
XLOC_037863	ACPP	3:132317366-132375444
XLOC_037866	DNAJC13	3:132417525-132539032
XLOC_037951	CHST2,RP11-80H8.4	3:143117890-143150921

XLOC_037964	PLSCR1	3:146502358-146544968
XLOC_037979	GYG1	3:148991340-149102823
XLOC_037988	RNF13	3:149761104-150225190
XLOC_038015	SUCNR1	3:151733915-151928175
XLOC_038032	MME	3:155024123-155183729
XLOC_038044	TIPARP	3:156671861-156706770
XLOC_038055	MLF1	3:158571162-158607252
XLOC_038056	GFM1,MFSD1,RP11-379F4.4	3:158644277-158831071
XLOC_038200	B3GNT5	3:183178042-183428778
XLOC_038258	EIF4A2,SNORA4,SNORA63,SNORA81,SNORD2	3:186781779-186807058
XLOC_038263	ST6GAL1	3:186930484-187078845
XLOC_038357	PAK2	3:196739842-196832647
XLOC_038376	FAM157A,RP11-694O4.1	3:198151970-198190392
XLOC_038396	SUMF1	3:3250686-4467281
XLOC_038479	WNT7A	3:13816257-13880452
XLOC_038566	SLC4A7	3:27372720-27484424
XLOC_038571	EOMES	3:27715947-27722711
XLOC_038595	CMTM6	3:32391586-32503408
XLOC_038602	GLB1,TMPPE	3:32996607-33097230
XLOC_038625	LRRFIP2	3:37052655-37183689
XLOC_038647	CSRNP1	3:39141854-39154562
XLOC_038649	CX3CR1	3:39232485-39281735
XLOC_038719	CCR1	3:46163603-46266706
XLOC_038723	LTF	3:46423648-46485234
XLOC_038726	ALS2CL	3:46668996-46693704
XLOC_038756	PFKFB4,UCN2	3:48517683-48563773
XLOC_038779	GPX1	3:49356891-49358600
XLOC_038780	AMT,NICN1,RHOA	3:49359070-49429604
XLOC_038802	NPRL2,RASSF1,ZMYND10	3:50329623-50504244
XLOC_038804	CACNA2D2	3:50329623-50504244
XLOC_038822	ABHD14B,PCBP4,RP11-155D18.12	3:51955313-51989197
XLOC_038826	ALAS1	3:52198085-52214327
XLOC_038844	TKT	3:53211813-53256069
XLOC_038867	ARHGEF3	3:56722981-57080101
XLOC_038877	ARF4	3:57571347-57600927
XLOC_038882	DNASE1L3	3:58192256-58214697
XLOC_038886	ACOX2	3:58505135-58537319
XLOC_038922	LRIG1	3:66133609-66501263
XLOC_038932	FRMD4B	3:69169989-69542642
XLOC_038945	PROK2	3:71770951-71785654
XLOC_038947	LINC00877	3:72033019-72242565
XLOC_039002	GBE1	3:81489698-81762161
XLOC_039059	FILIP1L	3:99817831-100179059
XLOC_039074	RPL24	3:101681090-101686782
XLOC_039084	CBLB	3:105655460-105870680
XLOC_039127	GCSAM	3:112086334-112133381
XLOC_039134	ATG3	3:112532501-112585577
XLOC_039226	HEG1	3:124965709-125055958
XLOC_039233	OSBPL11,Y_RNA	3:125528127-125595755

XLOC_039368	CLDN18,DZIP1L	3:137951457-138175109
XLOC_039372	CEP70	3:138494343-138594538
XLOC_039374	PIK3CB	3:138652698-138835003
XLOC_039410	TFDP2	3:141944338-142149544
XLOC_039412	GK5,RP11-485G4.2	3:142157331-142225609
XLOC_039420	PCOLCE2	3:142815921-142889236
XLOC_039438	PLSCR1,RP11-329N15.3	3:146502358-146544968
XLOC_039486	P2RY13	3:151085696-151458709
XLOC_039541	VEPH1	3:157143130-157533619
XLOC_039598	SERPINI2,WDR49	3:167441788-167653983
XLOC_039603	GOLIM4	3:168008676-168096059
XLOC_039622	EIF5A2	3:170888414-170908693
XLOC_039626	TNIK	3:171054012-171460446
XLOC_039629	PLD1	3:171600200-171810950
XLOC_039637	TNFSF10	3:172505507-172523507
XLOC_039640	NCEH1	3:172630248-172711218
XLOC_039684	GNB4,RP11-145M9.4	3:179347691-179451636
XLOC_039716	LAMP3	3:183122212-183163839
XLOC_039752	DGKG,ETV5	3:186046307-186362273
XLOC_039771	BCL6	3:187698258-187745727
XLOC_039823	XXYLT1	3:195067569-195271327
XLOC_039826	ACAP2	3:195274691-195443078
XLOC_039916	-	3:46416433-46418730
XLOC_040024	FGFRL1	4:1009923-1026899
XLOC_040074	D4S234E	4:4348139-4764361
XLOC_040080	STK32B	4:5051441-5500998
XLOC_040086	WFS1	4:6269848-6303265
XLOC_040089	AC093323.3	4:6670724-6676047
XLOC_040090	S100P	4:6679327-6697170
XLOC_040099	SORCS2	4:7192537-7742836
XLOC_040107	SH3TC1	4:8182071-8243878
XLOC_040189	BST1,FAM200B	4:15603883-15739411
XLOC_040198	LAP3	4:17577191-17614571
XLOC_040244	RBPJ	4:26163454-26435288
XLOC_040276	DTHD1,RP11-431M7.2	4:36281608-36392410
XLOC_040284	PGM2,RP11-177C12.1	4:37826181-37876353
XLOC_040288	LINC01259	4:38279010-38523180
XLOC_040295	FAM114A1	4:38867676-38945739
XLOC_040317	RHOH	4:40191052-40247732
XLOC_040331	LIMCH1	4:41359606-41700044
XLOC_040369	TXK	4:48063154-48269864
XLOC_040498	UBA6-AS1	4:67701269-68129880
XLOC_040552	SLC4A4	4:71186688-71572087
XLOC_040567	CXCL8	4:73740505-73743716
XLOC_040575	EREG	4:74365142-74388946
XLOC_040576	AREG	4:74445133-74455009
XLOC_040578	PARM1	4:74933094-75050115
XLOC_040591	FAM47E,FAM47E-STBD1	4:76158736-76311599
XLOC_040602	SEPT11	4:76949700-77040384
XLOC_040612	ANXA3	4:78551518-78610451

XLOC_040613	LINC01094	4:78617515-78682847
XLOC_040615	BMP2K	4:78776294-78939438
XLOC_040648	AGPAT9	4:83535761-83605875
XLOC_040656	ARHGAP24	4:85475113-86002811
XLOC_040733	DAPP1	4:99816739-99870484
XLOC_040767	TET2	4:105137279-105279816
XLOC_040781	SGMS2	4:107824562-107978799
XLOC_040785	LEF1-AS1	4:108047544-108341108
XLOC_040787	RPL34	4:108620461-108638681
XLOC_040820	C4orf32	4:112145396-112195256
XLOC_040823	ALPK1	4:112274541-112442620
XLOC_040864	USP53	4:119192772-119295517
XLOC_040998	SMAD1	4:145481193-145559148
XLOC_041051	RP11-18H21.1	4:152100753-152120145
XLOC_041067	RP11-153M7.5,TLR2	4:153682715-153961936
XLOC_041087	GUCY1A3,RP11-588K22.2	4:155666710-155737062
XLOC_041106	RP11-597D13.9,TMEM144	4:158124473-158255411
XLOC_041108	RP11-597D13.7	4:158262788-158280553
XLOC_041150	KLHL2	4:165070607-165323156
XLOC_041187	SAP30	4:173363779-173377532
XLOC_041207	NEIL3	4:177309835-177362943
XLOC_041230	STOX2	4:183853430-184023526
XLOC_041330	SPON2,TMED11P	4:1113638-1208962
XLOC_041357	LRPAP1,RP11-529E10.6	4:3503596-3532559
XLOC_041372	LYAR	4:4267700-4321786
XLOC_041385	JAKMIP1	4:6026198-6200591
XLOC_041391	MRFAP1L1	4:6707700-6709917
XLOC_041400	AFAP1	4:7754089-7940296
XLOC_041438	CLNK	4:10484856-10734856
XLOC_041466	FBXL5	4:15603883-15739411
XLOC_041470	FGFBP2,PROM1	4:15960240-16084378
XLOC_041474	LDB2	4:16400429-16898809
XLOC_041494	CDC42P6	4:22692913-22819575
XLOC_041503	CCDC149	4:24803655-24996149
XLOC_041542	ARAP2	4:35948220-36274220
XLOC_041547	RELL1,RP11-36B15.1	4:37453940-37686407
XLOC_041560	TLR1,TLR6	4:38744128-38856845
XLOC_041565	RPL9	4:39454123-39458948
XLOC_041581	RBM47	4:40423198-40631067
XLOC_041594	RP11-457P14.6	4:41896823-41934707
XLOC_041616	RAC1P2	4:46723829-46724408
XLOC_041623	NFXL1,RP11-121C2.2	4:47839858-48040173
XLOC_041625	TEC,TXK	4:48063154-48269864
XLOC_041631	OCIAD2	4:48885018-48906937
XLOC_041654	SGCB	4:52020705-52038482
XLOC_041690	HOPX	4:56647987-56681899
XLOC_041694	SPINK2	4:56809859-56821742
XLOC_041773	SULT1B1	4:69717372-69787961
XLOC_041777	IGJ	4:70430491-70686816
XLOC_041800	CXCL3	4:74036588-74038807

XLOC_041803	CXCL2	4:74097034-74099293
XLOC_041826	SCARB2	4:76158736-76311599
XLOC_041863	RASGEF1B,RP11-689K5.3	4:81426392-82044244
XLOC_041882	HPSE	4:83292460-83335153
XLOC_041893	WDFY3	4:84669083-85011277
XLOC_041909	HSD17B11,HSD17B13,RP11-529H2.2	4:87159936-87391386
XLOC_041957	TSPAN5	4:98470202-98658653
XLOC_042028	PAPSS1	4:107590275-107720452
XLOC_042032	LEF1	4:108047544-108341108
XLOC_042100	SEC24D	4:118722808-118838683
XLOC_042124	ANXA5	4:121667954-121697113
XLOC_042145	ANKRD50	4:124664051-124712732
XLOC_042221	SETD7	4:139495940-139606699
XLOC_042225	MAML3	4:139665681-140154184
XLOC_042234	TBC1D9	4:140619825-140758766
XLOC_042242	INPP4B	4:142023159-142847432
XLOC_042267	ZNF827	4:145680203-145938693
XLOC_042283	NR3C2	4:148078622-148445334
XLOC_042292	LRBA	4:150264442-151015745
XLOC_042339	MAP9	4:155276688-155381346
XLOC_042367	FAM198B	4:158124473-158255411
XLOC_042392	MARCH1	4:163494089-164384050
XLOC_042419	DDX60L	4:168356628-169010278
XLOC_042445	HMGB2,RP11-798M19.3	4:173131927-173335125
XLOC_042461	HPGD	4:174490176-174523154
XLOC_042510	CLDN22,CLDN24	4:183097016-183342811
XLOC_042528	ACSL1	4:184755512-184826818
XLOC_042530	RP11-701P16.5	4:184843403-184855751
XLOC_042602	-	4:15762542-15764792
XLOC_042678	-	4:158259726-158262041
XLOC_042760	CTD-2044J15.2,SRD5A1	5:6633342-6679761
XLOC_042821	CTD-2165H16.3,FAM105A	5:14581773-14641018
XLOC_042835	BASP1	5:17065597-17276843
XLOC_042941	RAI14	5:34656236-34832627
XLOC_042949	SPEF2	5:35617620-35827018
XLOC_042950	IL7R	5:35852694-35885081
XLOC_042957	SLC1A3	5:36606354-36725219
XLOC_042990	CARD6	5:40841183-40860175
XLOC_043053	SNX18	5:54517709-54554938
XLOC_043058	GZMK	5:54977863-55049253
XLOC_043063	GZMA	5:55102647-55110252
XLOC_043072	IL31RA	5:55851378-55922853
XLOC_043093	GAPT	5:58479644-58497246
XLOC_043107	ZSWIM6	5:61332267-61546671
XLOC_043115	IPO11,KIF2A,LRRC70	5:62306158-62629137
XLOC_043167	RP11-848G14.2,RP11-848G14.5	5:69607010-69637670
XLOC_043203	FCHO2	5:72955205-73090522
XLOC_043216	ARHGEF28	5:73626157-73941993
XLOC_043221	HEXB	5:74627405-74867854

XLOC_043243	F2R	5:76402482-76735781
XLOC_043244	F2RL1	5:76818781-76835693
XLOC_043283	ZFYVE16	5:80407632-80546912
XLOC_043284	FAM151B,RPL7P24	5:80407632-80546912
XLOC_043286	RASGRF2	5:80947696-81313297
XLOC_043303	VCAN	5:83471375-83588645
XLOC_043367	ARSK	5:95555073-95605255
XLOC_043373	AC008592.4,LINC01554	5:95829418-95860133
XLOC_043456	CAMK4	5:111223652-112017309
XLOC_043533	CTB-36H16.2,SNX2	5:122774982-122838178
XLOC_043561	LMNB1	5:126776622-126837069
XLOC_043588	CDC42SE2	5:131233022-131398802
XLOC_043625	TCF7	5:134091625-134371047
XLOC_043635	JADE2	5:134524311-134583295
XLOC_043655	TGFBI	5:136028894-136063870
XLOC_043679	EGR1	5:138465489-138469315
XLOC_043682	CTNNA1	5:138610966-138935034
XLOC_043703	IGIP,PURA	5:140072856-140129392
XLOC_043705	CYSTM1	5:140174641-140303192
XLOC_043760	ARHGAP26	5:142770375-143229011
XLOC_043801	ADRB2	5:148825244-149063163
XLOC_043815	PPARGC1B	5:149730290-149855022
XLOC_043824	NDST1	5:150475530-150558211
XLOC_043828	SMIM3	5:150777945-150796734
XLOC_043834	GM2A	5:151163711-151303766
XLOC_043837	SLC36A1	5:151437045-151497839
XLOC_043873	ITK	5:157080207-157255514
XLOC_043874	CYFIP2	5:157260121-157576108
XLOC_043907	MIR146A	5:160468267-160487426
XLOC_043992	CPEB4	5:173888279-173961976
XLOC_044012	CTC-251I16.1,HRH2	5:175657637-175710756
XLOC_044057	N4BP3	5:178113442-178126995
XLOC_044072	ZNF354C	5:179060377-179086270
XLOC_044107	BTNL9	5:181040224-181061523
XLOC_044115	AC138035.2	5:181306501-181324685
XLOC_044132	BRD9,CTD-2589H19.4,ZDHHC11,ZDHHC11B	5:710359-919357
XLOC_044149	AC091849.1,RP11-43F13.1,SDHAP3	5:1567420-1634225
XLOC_044233	DAP	5:10679229-10770294
XLOC_044245	-	5:14564470-14573058
XLOC_044250	ANKH	5:14661807-14871914
XLOC_044274	FTH1P10	5:17353694-17354899
XLOC_044395	IL7R	5:35852694-35885081
XLOC_044485	EMB	5:50337771-50443332
XLOC_044514	CTD-2313F11.1,ESM1	5:54977863-55049253
XLOC_044592	CTD-2116N20.1	5:65484341-65516599
XLOC_044605	CD180,CTD-2306M10.1	5:67179206-67196820
XLOC_044626	CCDC125,CFL1P5	5:69279316-69332852
XLOC_044637	RP11-1319K7.1	5:70094658-70128434
XLOC_044646	NAIP	5:70968482-71025114

XLOC_044694	GCNT4,LINC01336	5:75025193-75054103
XLOC_044715	S100Z	5:76849907-76921848
XLOC_044722	AP3B1,CTD-2179L22.1	5:77991487-78294755
XLOC_044726	LHFPL2	5:78485182-78770021
XLOC_044729	ARSB	5:78776769-78986087
XLOC_044734	JMY	5:78997605-79327394
XLOC_044751	ANKRD34B	5:80556753-80570488
XLOC_044776	TMEM167A	5:83052845-83353787
XLOC_044811	MEF2C	5:88507545-89466398
XLOC_044825	LUCAT1,RP11-213H15.4	5:91295970-91314486
XLOC_044843	MCTP1	5:94152965-95284575
XLOC_044901	ST8SIA4	5:100806314-100942091
XLOC_044979	TICAM2,TMED7,TMED7-TICAM2	5:115573653-115632992
XLOC_045033	CEP120	5:123344884-123423592
XLOC_045073	FBN2	5:128257908-129033642
XLOC_045089	P4HA2	5:132037322-132405223
XLOC_045095	KIF3A	5:132688680-132737638
XLOC_045098	SHROOM1	5:132822098-132830898
XLOC_045113	C5orf15	5:133955501-133968787
XLOC_045137	H2AFY	5:135027734-135401296
XLOC_045138	DCANP1,TIFAB	5:135444022-135458697
XLOC_045157	SPOCK1	5:136975297-137598379
XLOC_045173	ETF1	5:138506094-138543300
XLOC_045197	HBEGF	5:140332842-140346636
XLOC_045203	CD14	5:140618763-140633987
XLOC_045228	PCDH1	5:141850771-141879246
XLOC_045259	PPP2R2B,RNA5SP196	5:146563225-147084784
XLOC_045266	JAKMIP2	5:147585438-147782848
XLOC_045298	CSF1R	5:149960686-150113372
XLOC_045304	RPS14	5:150442818-150449756
XLOC_045451	SH3PXD2B	5:172325180-172454523
XLOC_045457	DUSP1	5:172574248-172778091
XLOC_045498	HK3	5:176810476-176899666
XLOC_045506	F12	5:177400102-177456286
XLOC_045509	DOK3	5:177456607-177511274
XLOC_045514	FAM153A	5:177682293-177794396
XLOC_045551	RNF130	5:179911650-180072118
XLOC_045564	FLT4	5:180601505-180649624
XLOC_045575	-	5:181186711-181190336
XLOC_045577	CTC-338M12.1,TRIM7	5:181191881-181217852
XLOC_045718	SERPINB1	6:2801444-2842368
XLOC_045735	FAM50B	6:3831932-3855737
XLOC_045762	LY86	6:6346464-6654985
XLOC_045765	RREB1	6:7107596-7251980
XLOC_045797	TMEM170B	6:11536991-11583524
XLOC_045806	RPL15P3	6:12514109-12514724
XLOC_045818	CD83	6:14117255-14136918
XLOC_045823	JARID2	6:15245480-15522040
XLOC_045848	RNF144B	6:18387230-18468874
XLOC_045856	E2F3	6:20401905-20493719

XLOC_045861	SOX4	6:21592768-21598619
XLOC_045931	BTN3A2	6:26365158-26378682
XLOC_045933	BTN3A1	6:26401935-26415387
XLOC_045935	BTN2A1,BTN3A3	6:26440226-26493769
XLOC_046074	DDR1,MIR4640	6:30876420-30900901
XLOC_046094	HCP5,MICA	6:31382809-31511124
XLOC_046103	TNF	6:31575478-31578336
XLOC_046105	AIF1	6:31615183-31617021
XLOC_046120	C2,CFB,XXbac-BPG116M5.17	6:31897784-31952084
XLOC_046127	EGFL8,PPT2,PPT2-EGFL8	6:32148358-32178096
XLOC_046137	-	6:32480011-32481928
XLOC_046167	PHF1	6:33410398-33418317
XLOC_046168	MIR5004,SYNGAP1,ZBTB9	6:33420069-33457611
XLOC_046171	ITPR3,MIR3934	6:33620247-33711948
XLOC_046180	PACSIN1	6:34466060-34535231
XLOC_046198	RPL10A	6:35468407-35470785
XLOC_046209	MAPK13,MAPK14	6:36027208-36145124
XLOC_046211	PNPLA1	6:36240803-36314220
XLOC_046213	KCTD20	6:36354410-36491232
XLOC_046219	CDKN1A	6:36676459-36687355
XLOC_046220	RAB44,RP3-431A14.4	6:36697786-36737986
XLOC_046223	FGD2	6:37005626-37153393
XLOC_046230	RP1-153P14.5,RP1-153P14.7,RP1-153P14.8	6:37507347-37571588
XLOC_046245	DAAM2	6:39792365-39934551
XLOC_046286	PTK7	6:43076267-43161719
XLOC_046305	VEGFA	6:43770183-43786487
XLOC_046313	SLC29A1	6:44219504-44234358
XLOC_046324	RUNX2	6:44806759-45666820
XLOC_046326	ENPP4	6:46129975-46146699
XLOC_046367	TMEM14A	6:52671108-52686588
XLOC_046369	RN7SK	6:52995619-52995950
XLOC_046417	FKBP1C	6:63211445-63213024
XLOC_046422	PTP4A1	6:63521760-63583587
XLOC_046454	OGFRL1	6:71221456-71420769
XLOC_046494	MYO6	6:75749184-75919963
XLOC_046531	CYB5R4	6:83859642-83971383
XLOC_046540	NT5E	6:85449583-85495791
XLOC_046556	C6orf165,SLC35A1	6:87407982-87512883
XLOC_046581	CASP8AP2	6:89829892-89874436
XLOC_046689	FOXO3	6:108559783-108775655
XLOC_046704	FIG4,RP1-249I4.2	6:109691274-109829368
XLOC_046719	SLC16A10	6:111087502-111259034
XLOC_046742	MARCKS	6:113857361-113863471
XLOC_046748	DSE,RP1-93H18.1,RP1-93H18.7,RP3-486I3.7	6:116249960-116445076
XLOC_046755	KPNA5	6:116681095-116741893
XLOC_046794	SMPDL3A	6:122789048-122809720
XLOC_046799	RNF217	6:124909092-125092633
XLOC_046806	RP11-527F13.1,TRMT11	6:125986402-126248048

XLOC_046827	LAMA2	6:128883140-129516569
XLOC_046836	ARG1	6:131572584-131628373
XLOC_046860	RPS12	6:132814440-132847717
XLOC_046884	MYB	6:135181299-135219173
XLOC_046889	PDE7B	6:135851649-136225595
XLOC_046904	TNFAIP3	6:137823672-137883312
XLOC_046947	STX11	6:144147938-144191960
XLOC_046957	RAB32	6:146543692-146554965
XLOC_046966	SASH1	6:148237584-148552050
XLOC_046970	UST	6:148746797-149077318
XLOC_046977	GINM1	6:149566293-149592663
XLOC_047082	IGF2R,RP11-288H12.4	6:159969035-160158718
XLOC_047145	-	6:170464605-170474167
XLOC_047156	EXOC2	6:485015-693138
XLOC_047172	SERPINB1	6:2801444-2842368
XLOC_047175	SERPINB9	6:2885024-2903889
XLOC_047216	F13A1	6:6144072-6321171
XLOC_047289	-	6:17585289-17599036
XLOC_047294	KIF13A	6:17759114-17987657
XLOC_047380	GUSBP2	6:26826422-26956958
XLOC_047389	ZNF204P	6:27357656-27403904
XLOC_047491	FLOT1	6:30727708-30742869
XLOC_047493	IER3	6:30742928-30744575
XLOC_047533	CLIC1,DDAH2	6:31727037-31739763
XLOC_047581	DAXX	6:33318557-33329269
XLOC_047623	FKBP5	6:35570087-35728583
XLOC_047629	SLC26A8	6:35943513-36024868
XLOC_047631	RP1-179N16.3	6:36027208-36145124
XLOC_047654	CCDC167	6:37482919-37499922
XLOC_047670	KCNK5	6:39188972-39229450
XLOC_047689	RP1-229K20.5,TREM1,TREML3P	6:41208592-41291474
XLOC_047747	ENPP5	6:46159072-46170989
XLOC_047751	PLA2G7	6:46652914-46735693
XLOC_047771	CRISP3	6:49727375-49744437
XLOC_047790	MCM3	6:52263888-52284881
XLOC_047953	ELOVL4	6:79914811-79947580
XLOC_047961	FAM46A	6:81491438-81752774
XLOC_048023	GABRR2,UBE2J1	6:89254478-89353161
XLOC_048024	RRAGD	6:89364635-89412270
XLOC_048094	CD24	6:106969830-106975627
XLOC_048100	RP1-47M23.3,SCML4	6:107695422-107824457
XLOC_048106	SNX3	6:108211221-108261260
XLOC_048125	WASF1	6:110099818-110180004
XLOC_048145	FYN	6:111660331-111873630
XLOC_048198	MAN1A1	6:119177208-119349861
XLOC_048237	THEMIS	6:127700178-127952643
XLOC_048254	RP11-73O6.3,SAMD3	6:130013698-130443241
XLOC_048270	STX7	6:132445297-132611185
XLOC_048277	VNN1	6:132681589-132714049
XLOC_048279	VNN2	6:132743869-132763483

XLOC_048290	SGK1	6:134169245-134318112
XLOC_048326	IFNGR1	6:137197483-137219449
XLOC_048333	PERP	6:138088504-138107511
XLOC_048360	HIVEP2,RP11-439L18.2	6:142748442-142958052
XLOC_048370	PLAGL1	6:143940299-144064753
XLOC_048438	SYNE1	6:151656690-152637801
XLOC_048487	TAGAP	6:158948086-159065273
XLOC_048494	SOD2	6:159669056-159798436
XLOC_048501	LPAL2	6:160348267-160520841
XLOC_048575	-	6:170464605-170474167
XLOC_048652	-	6:49718061-49719271
XLOC_048684	-	6:119349927-119356978
XLOC_048747	FAM20C	7:182934-352156
XLOC_048778	AC004840.9,CHST12	7:2403451-2463073
XLOC_048782	TTYH3	7:2631940-2664802
XLOC_048818	ZNF853	7:6615616-6624290
XLOC_048880	AHR	7:17279833-17346152
XLOC_048921	AC005082.12	7:23206012-23208045
XLOC_048937	MPP6	7:24573267-24694275
XLOC_048943	-	7:26101642-26126737
XLOC_048946	SNX10	7:26291891-26376701
XLOC_048970	CREB5	7:28299320-28826165
XLOC_049014	FKBP9	7:32957403-33007205
XLOC_049040	ANLN	7:36324220-36724567
XLOC_049136	UPP1	7:48070312-48108733
XLOC_049137	ABCA13	7:48171433-48647496
XLOC_049332	AUTS2	7:69598918-70793068
XLOC_049379	NCF1	7:74773674-74791579
XLOC_049406	HSPB1	7:76302543-76304295
XLOC_049431	MAGI2-AS3	7:78017056-79471208
XLOC_049436	CD36	7:80330191-80679835
XLOC_049462	ADAM22	7:87934142-88202956
XLOC_049482	FZD1	7:91264363-91271326
XLOC_049542	LMTK2	7:98106870-98209633
XLOC_049544	BRI3	7:98211426-98401068
XLOC_049578	PVRIG,STAG3	7:100177562-100272865
XLOC_049586	AGFG2	7:100539188-100568219
XLOC_049613	SH2B2	7:102284618-102321711
XLOC_049653	CDHR3	7:105876766-106034061
XLOC_049683	LRRN3	7:110663050-111562517
XLOC_049710	CAPZA2	7:116811069-116922049
XLOC_049798	IRF5	7:128937268-129055203
XLOC_049810	SMKR1	7:129502478-129512932
XLOC_049811	-	7:129525463-129533825
XLOC_049864	NUP205	7:135557918-135648849
XLOC_049905	TBXAS1	7:139561569-140063721
XLOC_049930	MGAM	7:141704286-142107008
XLOC_049935	TRBV1	7:142298796-142299897
XLOC_049936	TRBV2	7:142299999-142301439
XLOC_049937	TRBV3-1	7:142308201-142309587

XLOC_049938	TRBV4-1	7:142313183-142313666
XLOC_049939	TRBV5-1	7:142320315-142321629
XLOC_049940	TRBV6-1	7:142328296-142328786
XLOC_049942	TRBV4-2	7:142345420-142345985
XLOC_049943	-	7:142348776-142350068
XLOC_049944	-	7:142350456-142353406
XLOC_049951	TRBV9	7:142391890-142392412
XLOC_049957	TRBV6-5	7:142435822-142452425
XLOC_049960	TRBV6-6	7:142469192-142470387
XLOC_049964	TRBV5-6	7:142499130-142501103
XLOC_049968	TRBV7-9	7:142529289-142529762
XLOC_049970	TRBV10-3	7:142543004-142544926
XLOC_049972	TRBV12-3	7:142560422-142560931
XLOC_049973	TRBV12-4	7:142563425-142564266
XLOC_049975	TRBV14	7:142587867-142588359
XLOC_049976	TRBV15	7:142592927-142593473
XLOC_049979	TRBV18,TRBV19	7:142615552-142621378
XLOC_049980	TRBV20-1	7:142624560-142627421
XLOC_049983	TRBV23-1	7:142645960-142646467
XLOC_049986	PGBD4P1,TRBC2,TRBJ2-1,TRBJ2-2,TRBJ2-2P,TRBJ2-3,TRBJ2-5,TRBJ2-6,TRBJ2-7,TRBV25-1,TRBV27,TRBV28	7:142670669-142803938
XLOC_050008	ZYX	7:143379340-143529691
XLOC_050077	RP4-800G7.2,ZNF783	7:149261997-149297312
XLOC_050087	ATP6V0E2	7:149867696-150076655
XLOC_050096	GIMAP7	7:150514829-150521073
XLOC_050102	GIMAP1,GIMAP5,RP5-1051J4.4	7:150716359-150751528
XLOC_050145	-	7:155263322-155266978
XLOC_050173	AC011899.9,RP11-452C13.1	7:157539055-158587788
XLOC_050183	-	7:13594-36896
XLOC_050198	MICALL2	7:1428464-1459518
XLOC_050218	CARD11	7:2904846-3043979
XLOC_050220	-	7:3093515-3118095
XLOC_050240	AC006483.1,ACTB	7:5525056-5563784
XLOC_050291	ICA1	7:8113183-8344516
XLOC_050381	DFNA5	7:24698352-24758113
XLOC_050382	OSBPL3	7:24796538-24981634
XLOC_050400	SKAP2	7:26667061-26995239
XLOC_050421	CREB5	7:28299320-28826165
XLOC_050423	CPVL	7:28979966-29563670
XLOC_050484	AOAH	7:36324220-36724567
XLOC_050493	AMPH,RP11-121A8.1,TRGC1,TRGC2,TRGJ1,TRGJ2,TRGJP,TRGJP1,TRGJP2,TRGV1,TRGV2,TRGV3,TRGV4,TRGV5,TRGV5P,TRGV6,TRGV7,TRGV8,TRGV9,TRGVA	7:38239518-38631567
XLOC_050519	INHBA	7:41667167-41779388
XLOC_050561	IGFBP3	7:45912244-45921874
XLOC_050569	TNS3	7:47275149-47582558

XLOC_050586	FIGNL1	7:50436114-50565457
XLOC_050588	GRB10	7:50590062-50793462
XLOC_050737	GUSB	7:65960683-65982314
XLOC_050824	NCF1	7:74773674-74791579
XLOC_050830	NCF1C	7:75156638-75172044
XLOC_050841	HIP1	7:75533084-75739088
XLOC_050852	YWHAG	7:76326793-76359031
XLOC_050866	FGL2	7:76959801-77416646
XLOC_050881	AC073850.6	7:80330191-80679835
XLOC_050882	CD36	7:80330191-80679835
XLOC_050883	SEMA3C	7:80742537-80922359
XLOC_050890	HGF	7:81699005-81770487
XLOC_050908	KIAA1324L	7:86876905-87059699
XLOC_050912	TP53TG1	7:87324690-87345550
XLOC_050914	ABCB1	7:87502928-87832296
XLOC_050915	SLC25A40	7:87833556-87909541
XLOC_050917	CTB-167B5.2,STEAP4	7:88203521-88307019
XLOC_050965	PDK4	7:95583498-95619940
XLOC_051078	DPY19L2P2	7:103175132-103280416
XLOC_051095	NAMPT	7:106248284-106286359
XLOC_051112	NRCAM	7:108147622-108456717
XLOC_051125	DOCK4	7:111726109-112206463
XLOC_051149	TFEC	7:115935147-116159896
XLOC_051197	AASS	7:122073546-122144280
XLOC_051234	PRRT4	7:128350324-128362099
XLOC_051288	PLXNA4	7:132123331-132648688
XLOC_051299	AKR1B1	7:134442349-134459284
XLOC_051360	KIAA1147,RP5-894A10.2,RP5-894A10.6	7:141529202-141702163
XLOC_051368	CLEC5A	7:141704286-142107008
XLOC_051380	TRBC2	7:142670669-142803938
XLOC_051402	RP11-61L23.2	7:143761789-143836933
XLOC_051429	PDIA4	7:149003053-149028641
XLOC_051437	ZNF777	7:149431362-149461123
XLOC_051440	ZNF467	7:149764171-149833979
XLOC_051455	GIMAP6	7:150624011-150673590
XLOC_051458	-	7:150759372-150781181
XLOC_051459	TMEM176B	7:150791284-150805120
XLOC_051461	ATG9B	7:150990994-151024499
XLOC_051470	SMARCD3	7:151231637-151277896
XLOC_051556	INMT	7:30651942-30925516
XLOC_051641	-	7:150757051-150759283
XLOC_051656	ARHGEF10	8:1823975-1958641
XLOC_051658	KBTBD11	8:1971396-2006936
XLOC_051661	MYOM2	8:2045039-2491307
XLOC_051663	MYOM2	8:2045039-2491307
XLOC_051727	-	8:8920093-8927548
XLOC_051741	MSRA	8:10054267-10428954
XLOC_051841	LPL	8:19901716-19967258
XLOC_051843	ATP6V1B2,RNU6-892P	8:20197341-20233342

XLOC_051865	SLC39A14	8:22367248-22434129
XLOC_051867	PPP3CC,SORBS3	8:22440818-22575788
XLOC_051875	TNFRSF10C	8:23084354-23122083
XLOC_051895	DOCK5	8:25184672-25418082
XLOC_051902	BNIP3L	8:26379258-26514092
XLOC_051905	DPYSL2	8:26514274-26658178
XLOC_051914	EPHX2	8:27490778-27615031
XLOC_051970	NRG1,RP11-1002K11.1	8:31639385-32767959
XLOC_052022	-	8:38215350-38224765
XLOC_052033	ADAM9	8:38988807-39105144
XLOC_052166	LYN,RP11-446E9.2	8:55876698-56025350
XLOC_052185	FAM110B	8:57994508-58204279
XLOC_052188	SDCBP	8:58552923-58661826
XLOC_052241	C8orf44,C8orf44-SGK3,SGK3	8:66667454-66862022
XLOC_052295	LY96	8:73991204-74037298
XLOC_052317	IL7,ZC2HC1A	8:78666046-78941769
XLOC_052375	CPNE3	8:86333273-86765023
XLOC_052385	RIPK2	8:89609408-89791298
XLOC_052438	SDC2	8:96493350-96611780
XLOC_052439	CPQ	8:96645226-97149654
XLOC_052502	KB-1507C5.4	8:102978784-103002424
XLOC_052504	ATP6V1C1	8:103020186-103073364
XLOC_052509	BAALC	8:103121031-103333114
XLOC_052591	ZHX2	8:122781393-122974512
XLOC_052611	FAM91A1	8:123768442-123815523
XLOC_052630	TRIB1	8:125430047-125438416
XLOC_052709	DENND3	8:141117277-141196050
XLOC_052715	PTP4A3	8:141389938-141432598
XLOC_052737	LY6E	8:142981737-143032629
XLOC_052745	RHPN1	8:143368815-143384220
XLOC_052759	CTD-3065J16.6,SPATC1	8:143856161-144063832
XLOC_052844	DEFA4	8:6934847-6938368
XLOC_052850	DEFA1B,DEFA3	8:6996765-7018301
XLOC_052892	ALG1L13P	8:8228594-8245441
XLOC_052893	SGK223	8:8317729-8386498
XLOC_052937	CTSB	8:11842065-11869448
XLOC_052991	MSR1	8:16107773-16567490
XLOC_053001	MTUS1	8:17576432-17800917
XLOC_053004	ASAH1	8:18034099-18096394
XLOC_053029	REEP4	8:22138019-22141955
XLOC_053084	CLU,MIR6843	8:27490778-27615031
XLOC_053092	NUGGC	8:28020876-28084110
XLOC_053122	GSR	8:30678060-30727926
XLOC_053170	RAB11FIP1	8:37858617-37899467
XLOC_053207	ZMAT4	8:40530428-40897833
XLOC_053265	CEBPD	8:47260574-47739086
XLOC_053306	LYPLA1	8:54046366-54102017
XLOC_053321	RPS20,SNORD54	8:56066740-56074581
XLOC_053325	PLAG1	8:56160903-56211327
XLOC_053331	LINC00968	8:56436673-56559823

XLOC_053346	TOX	8:58797386-59121346
XLOC_053364	ASPH	8:61057157-61714647
XLOC_053387	RP11-1D12.2	8:64800623-64817590
XLOC_053396	MYBL1	8:66562174-66614961
XLOC_053400	SNHG6,SNORD87	8:66870748-66926398
XLOC_053455	RP11-463D19.2,STAU2,UBE2W	8:73420003-73878910
XLOC_053582	TMEM55A	8:90990706-91041064
XLOC_053596	TRIQK	8:92882983-93017673
XLOC_053611	FSBP,RAD54B,RP11-267M23.1	8:94371959-94475113
XLOC_053644	RPL30	8:97868839-98047353
XLOC_053651	STK3	8:98401402-98942827
XLOC_053698	NCALD	8:101686542-102124907
XLOC_053700	RRM2B	8:102204501-102239311
XLOC_053704	KLF10	8:102646461-102655973
XLOC_053707	AZIN1	8:102826356-102977876
XLOC_053774	TRPS1	8:115408432-115809673
XLOC_053781	EXT1	8:117794489-118111899
XLOC_053845	-	8:125859607-125877548
XLOC_053846	LINC00861,SOD1P3	8:125916485-125955412
XLOC_053860	-	8:127663183-127674890
XLOC_053875	FAM49B	8:129832300-130017129
XLOC_053879	ASAP1	8:130052103-130443660
XLOC_053893	SLA	8:132866957-133134903
XLOC_054070	-	8:89380821-89380982
XLOC_054144	JAK2	9:4984456-5129917
XLOC_054224	CNTLN	9:17134981-17503923
XLOC_054322	ANXA2P2	9:33624273-33625293
XLOC_054333	UBE2R2	9:33750465-33920404
XLOC_054349	GALT,IL11RA	9:34638132-34682302
XLOC_054361	RUSC2	9:35489855-35563899
XLOC_054367	CCDC107	9:35658265-35681159
XLOC_054383	GLIPR2	9:36136314-36164531
XLOC_054412	SNX18P3	9:38540561-38624990
XLOC_054600	ANXA1	9:73151756-73170393
XLOC_054618	GCNT1	9:76419849-76906087
XLOC_054630	VPS13A	9:77176755-77421541
XLOC_054636	CEP78	9:78234588-78293301
XLOC_054694	DAPK1	9:87497227-87708754
XLOC_054697	CTSL	9:87725518-87731393
XLOC_054714	C9orf47,S1PR3	9:88990862-89005011
XLOC_054721	-	9:89526943-89577548
XLOC_054722	GADD45G	9:89605012-89606790
XLOC_054730	SYK	9:90801497-90902297
XLOC_054757	C9orf89	9:93096167-93113283
XLOC_054772	HIATL1	9:94374334-94461042
XLOC_054803	HABP4	9:96450171-96619830
XLOC_054832	GALNT12	9:98807637-98872415
XLOC_054876	NIPSNAP3A,NIPSNAP3B	9:104747668-104777764
XLOC_054879	SLC44A1	9:105244515-105439171
XLOC_054896	RAD23B	9:107283136-107332399

XLOC_054918	DNAJC25,DNAJC25-GNG10,GNG10	9:111631304-111670246
XLOC_054921	UGCG	9:111896765-111935369
XLOC_054930	RP11-408O19.5,SNX30	9:112750753-112899831
XLOC_054932	SLC31A2	9:113150646-113221459
XLOC_054933	SLC31A1	9:113221561-113293978
XLOC_054937	RGS3	9:113444730-113597743
XLOC_054961	TRIM32	9:116424985-117415070
XLOC_054966	TLR4	9:117704202-117769444
XLOC_054980	GSN	9:121074204-121332843
XLOC_055018	NEK6	9:124257605-124415478
XLOC_055049	ZBTB34	9:126860629-126885878
XLOC_055057	-	9:127579283-127592083
XLOC_055059	C9orf117,TTC16	9:127612264-127736622
XLOC_055066	LCN2	9:128149070-128153474
XLOC_055076	CERCAM	9:128411750-128437497
XLOC_055083	SPTAN1	9:128552460-128656787
XLOC_055091	LRRC8A	9:128818473-128918066
XLOC_055099	-	9:129236190-129239050
XLOC_055103	C9orf106	9:129321015-129327203
XLOC_055108	LINC00963,RP11-492E3.51	9:129483450-129513686
XLOC_055115	USP20	9:129827289-129882271
XLOC_055155	EEF1A1P5	9:133019485-133020874
XLOC_055175	RXRA	9:134317097-134441499
XLOC_055221	LCN12	9:136791322-136957733
XLOC_055223	LCNL1,PTGDS,RP11-229P13.19	9:136974856-136986410
XLOC_055224	C9orf142	9:136992370-136994027
XLOC_055242	TOR4A	9:137277748-137282641
XLOC_055253	FAM157B	9:138216186-138252994
XLOC_055262	CBWD1	9:119810-179147
XLOC_055289	SPATA6L	9:4490443-4666788
XLOC_055312	RANBP6	9:6010677-6015663
XLOC_055353	TTC39B	9:15163621-15307360
XLOC_055355	PSIP1	9:15422703-15511328
XLOC_055361	BNC2	9:16409502-16871007
XLOC_055373	PLIN2	9:19108374-19149290
XLOC_055438	C9orf72,MOB3B	9:27325208-27573866
XLOC_055441	LINGO2	9:27947518-29213876
XLOC_055473	AQP3	9:33441153-33447611
XLOC_055520	FAM214B	9:35104111-35116341
XLOC_055526	SIT1	9:35649294-35650950
XLOC_055527	RMRP	9:35657750-35658018
XLOC_055529	TPM2	9:35681991-35691152
XLOC_055571	CNTNAP3,RP11-290L7.3	9:39071986-39288478
XLOC_055658	RNA5SP283	9:62798834-62813501
XLOC_055750	LINC01504,ZFAND5	9:72305228-72365402
XLOC_055779	GNAQ	9:77716086-78031458
XLOC_055805	FRMD3	9:83242989-83538546
XLOC_055813	SLC28A3	9:84275456-84368739
XLOC_055815	AGTPBP1	9:85541302-85881460

XLOC_055837	CDK20	9:87966155-87974766
XLOC_055863	RP11-305L7.1,RP11-305L7.3,RP11-367F23.1,RP11-367F23.2	9:90955882-91182762
XLOC_055868	NFIL3	9:91409039-91424642
XLOC_055891	NINJ1	9:93121488-93134288
XLOC_055904	FBP1	9:94603132-94640249
XLOC_055912	PTCH1	9:95442979-95517057
XLOC_055944	HEMGN	9:97926790-97944856
XLOC_055947	CORO2A	9:98120974-98192679
XLOC_055948	TBC1D2	9:98198983-98255830
XLOC_055973	MRPL50	9:101387632-101398645
XLOC_055994	ABCA1	9:104780740-104928892
XLOC_056012	KLF4	9:107484667-107752466
XLOC_056041	TXN	9:110243810-110256640
XLOC_056047	LPAR1	9:110668770-111039090
XLOC_056075	FKBP15	9:113150646-113221459
XLOC_056117	MEGF9	9:120600812-120714977
XLOC_056126	STOM	9:121338987-121370304
XLOC_056162	NR6A1	9:124517274-124772927
XLOC_056164	RPL35	9:124853416-124862012
XLOC_056188	FAM129B	9:127505298-127579030
XLOC_056195	AK1,RP11-203J24.9,ST6GALNAC4,ST6GALNAC6	9:127864857-127917065
XLOC_056199	FAM102A	9:127934502-127981066
XLOC_056227	LINC01503	9:129330656-129359538
XLOC_056236	PTGES	9:129738330-129777367
XLOC_056250	FAM78A	9:131257851-131278011
XLOC_056268	RALGDS	9:133097718-133149334
XLOC_056269	GBGT1	9:133149983-133163951
XLOC_056277	SLC2A6	9:133459964-133479517
XLOC_056281	DBH-AS1,SARDH	9:133636359-133739955
XLOC_056282	VAV2	9:133761893-133992826
XLOC_056289	FCN1	9:134641773-134918129
XLOC_056312	NACC2	9:136006331-136095297
XLOC_056323	INPP5E	9:136428582-136439892
XLOC_056330	AGPAT2	9:136673142-136687467
XLOC_056335	LCN8	9:136754384-136758543
XLOC_056344	CLIC3	9:136994633-136996916
XLOC_056345	ABCA2,FUT7,NPDC1,RP11-229P13.27	9:137007226-137046218
XLOC_056347	SAPCD2	9:137057662-137071092
XLOC_056363	MIR7114,NSMF,PNPLA7	9:137447328-137550852
XLOC_056366	ZMYND19	9:137582078-137590490
XLOC_056401	-	9:41241310-41242507
XLOC_056409	-	9:62818034-62819171
XLOC_060335	-	KI270745.1:21762-22985
XLOC_060398	CLCN4	X:10156636-10237662
XLOC_060410	TLR7	X:12866969-12890531
XLOC_060411	TLR8	X:12902816-12925987
XLOC_060429	MOSPD2	X:14873265-14923663

XLOC_060430	BMX	X:15384798-15556529
XLOC_060469	PHEX	X:22032319-22252442
XLOC_060486	SAT1	X:23783065-23786545
XLOC_060537	GK	X:30653358-30733160
XLOC_060576	CYBB	X:37349274-38688920
XLOC_060598	ATP6AP2	X:40580907-40606674
XLOC_060640	RP2	X:46836797-46899703
XLOC_060649	USP11	X:47232689-47248328
XLOC_060655	TIMP1	X:47557224-47619853
XLOC_060694	SUV39H1	X:48695553-48737163
XLOC_060710	PLP2	X:49171487-49186528
XLOC_060785	TSPYL2	X:53081164-53088540
XLOC_060813	MAGEH1	X:55452104-55453566
XLOC_060851	MIR223	X:65923534-66040125
XLOC_060886	DLG3	X:70444860-70505490
XLOC_060905	NHSL2,RP11-262D11.1,RP11-262D11.2	X:72069671-72161750
XLOC_060921	CHIC1	X:73563145-73687111
XLOC_060959	MAGEE1	X:76427680-76431353
XLOC_060969	PGK1	X:78065187-78139713
XLOC_060974	GPR174	X:79144396-79175417
XLOC_060978	-	X:79444389-79457154
XLOC_060989	SH3BGRL	X:81113700-81405628
XLOC_061046	DIAPH2	X:96684621-97642589
XLOC_061092	GPRASP1	X:102599167-102717733
XLOC_061146	FRMPD3	X:107449976-107605420
XLOC_061147	PRPS1	X:107628423-107651032
XLOC_061151	MID2	X:107825730-107935980
XLOC_061153	VSIG1	X:108018859-108079210
XLOC_061228	IL13RA1	X:118727571-118794605
XLOC_061315	XPNPEP2	X:129738973-129769625
XLOC_061360	SMIM10	X:134990937-134992473
XLOC_061380	FHL1	X:136147399-136211359
XLOC_061389	CD40LG	X:136647895-136662068
XLOC_061459	AFF2	X:148500618-149000663
XLOC_061484	HMGB3	X:150980508-150990775
XLOC_061508	ZNF185	X:152914441-152973480
XLOC_061518	ZNF275	X:153334154-153360110
XLOC_061544	TKTL1	X:154257537-154330363
XLOC_061590	P2RY8	X:1454037-1537749
XLOC_061664	GPX1P1	X:13377187-13419593
XLOC_061686	AP1S2	X:15789806-15855891
XLOC_061777	CXorf21	X:30553222-30578295
XLOC_061783	DMD	X:31097676-33339441
XLOC_061847	EFHC2	X:44146862-44343907
XLOC_061856	MIR221,MIR222,MIR222HG	X:45745210-45770274
XLOC_061888	CFP	X:47621572-47630781
XLOC_061909	SLC38A5	X:48458536-48470256
XLOC_061920	PIM2	X:48913092-48919566
XLOC_061923	TFE3	X:49028718-49043486

XLOC_062035	ARHGEF9	X:63634966-63809274
XLOC_062054	VSIG4	X:65923534-66040125
XLOC_062067	PJA1	X:69160814-69165793
XLOC_062094	CXorf65,IL2RG	X:71095873-71114013
XLOC_062106	CXCR3	X:71615915-71618885
XLOC_062180	ITM2A	X:79360382-79367667
XLOC_062229	EIF4A1P10	X:91779260-92623230
XLOC_062235	NAP1L3	X:93670929-93673568
XLOC_062272	BTK,TIMM8A	X:101341458-101390796
XLOC_062289	BEX5	X:102153704-102156057
XLOC_062306	BEX2	X:103309345-103311046
XLOC_062330	MORC4	X:106802679-107000244
XLOC_062347	ACSL4,KCNE1L	X:109623699-109734139
XLOC_062424	LAMP2	X:120425965-120469407
XLOC_062612	LDOC1	X:141175744-141177125
XLOC_062624	SLITRK4	X:143622789-143635777
XLOC_062713	PDZD4	X:153802165-153830661
XLOC_062716	RENBP	X:153935262-153944700
XLOC_062735	G6PD	X:154531341-154567967
XLOC_062743	MPP1	X:154778683-154821007
XLOC_062752	RAB39B	X:155258240-155264589
XLOC_062778	-	X:12927273-12930183
XLOC_062796	-	X:30608027-30611046
XLOC_062801	-	X:39178402-39180490
XLOC_062837	-	X:74571960-74573754
XLOC_062895	LINC00278	Y:2934405-3200160
XLOC_062920	PRKY,RNU6-941P	Y:7273694-7382117

Supplemental Table S4. List of 27 differentially expressed transcripts and their corresponding genes in the patients with HBV-ACLF compared with those with CHB.

Isoform ID	Gene ID	Gene Name	TSS group ID	Nearest_ref_ID	Locus	Length
TCONS_00014582	XLOC_002891	SDHB	TSS7459	ENST00000375499	1:17018721-17054170	1153
TCONS_00015513	XLOC_003054	PTAFR	TSS7950	ENST00000539896	1:28147165-28234402	3975
TCONS_00020279	XLOC_004017	CH17-385C13.2	TSS10353	ENST00000618406	1:146312887-146370251	9698
TCONS_00042970	XLOC_009130	ATM	TSS22382	ENST00000452508	11:108151494-108467531	12954
TCONS_00044024	XLOC_009279	CRTAM	TSS22907	ENST00000227348	11:122838383-122872639	2469
TCONS_00058643	XLOC_012022	RP11-693J15.5	TSS30208	ENST00000504409	12:92420093-92492091	10626
TCONS_00062349	XLOC_012657	CLEC2B	TSS32048	ENST00000538152	12:9827309-9870830	27398
TCONS_00102641	XLOC_020945	RPL13	TSS53093	ENST00000567815	16:89560468-89597246	712
TCONS_00136668	XLOC_026940	LRP3	TSS70319	ENST00000253193	19:33177602-33225850	3807
TCONS_00137320	XLOC_027009	CAPNS1	TSS70590	ENST00000588815	19:36139573-36150741	1471
TCONS_00138530	XLOC_027144	RPS19	TSS71144	ENST00000221975	19:41859698-41872926	500
TCONS_00147596	XLOC_028409	MAP4K1	TSS75043	ENST00000396857	19:38587640-38618882	2653

TCONS_00170726	XLOC_032760	RP11-378A13.2	TSS86676	ENST00000608367	2:218381687-218406424	24465
TCONS_00181187	XLOC_035353	TMEM50B	TSS92268	ENST00000420455	21:33402895-33480011	12947
TCONS_00183517	XLOC_035885	MIAT	TSS93496	ENST00000620145	22:26646427-26921033	10069
TCONS_00184629	XLOC_036064	APOBEC3A	TSS94011	ENST00000402255	22:38952455-38998209	6406
TCONS_00194486	XLOC_037859	NUDT16	TSS98913	ENST00000521288	3:131381670-131389623	6108
TCONS_00198534	XLOC_038620	TRANK1	TSS101048	ENST00000429976	3:36819095-36945057	10481
TCONS_00215933	XLOC_042316	TMEM154	TSS110109	ENST00000304385	4:152617478-152680165	10698
TCONS_00235621	XLOC_046948	UTRN	TSS120597	ENST00000367545	6:144284836-144853034	12425
TCONS_00246968	XLOC_049519	AC002451.3	TSS126630	ENST00000432265	7:95583498-95619940	20175
TCONS_00251229	XLOC_050363	TOMM7	TSS128884	ENST00000358435	7:22812627-22822852	778
TCONS_00258384	XLOC_051885	SLC25A37	TSS132581	ENST00000518881	8:23528596-23575645	7198
TCONS_00259677	XLOC_052186	UBXN2B	TSS133322	ENST00000399598	8:58411241-58452145	5068
TCONS_00261421	XLOC_052634	RNU6-442P	TSS134324	ENST00000383968	8:125895805-125909245	13440
TCONS_00266684	XLOC_053851	FAM84B	TSS137105	ENST00000304916	8:126552441-127021014	5404
TCONS_00273384	XLOC_055383	MLLT3	TSS140735	ENST00000380338	9:20341664-20622543	6772

Supplemental Table S5. List of the top 10 upregulated and downregulated transcripts (P<0.0001) and their corresponding genes in the patients with HBV-ACLF compared with those with CHB.

Isoform ID	Gene Name	Fold change_ log2 (ACLF/CHB)	
		Isoform	Gene
TCONS_00035981	NA	NA	NA
TCONS_00243180	NA	NA	NA
TCONS_00208306	CXCL8	6.9	5.8
TCONS_00051341	MMP8	6.9	6.5
TCONS_00138664	CD177	6.4	6.5
TCONS_00199080	LTF	5.7	5.7
TCONS_00132595	PRTN3	5.7	5.7
TCONS_00081046	CTSG	5.7	5.5
TCONS_00271603	LCN2	5.4	5.3
TCONS_00174008	BPI	5.3	5.3
TCONS_00074560	NA	NA	NA
TCONS_00017527	SLC1A7	-3.8	-3.9
TCONS_00236471	NA	-3.7	-3.2
TCONS_00211564	SPON2	-3.5	-3.2
TCONS_00294225	TKTL1	-3.3	-2.5
TCONS_00015345	ZNF683	-3.2	-3.0
TCONS_00155289	GNLY	-3.1	-2.7
TCONS_00008906	FCRL6	-3.1	-2.3
TCONS_00098313	SBK1	-3.1	-2.7
TCONS_00100183	GPR56	-3.1	-3.2
